# Peristaltic Waves as Optimal Gaits in Metameric Bio-Inspired Robots

**DOI:** 10.3389/frobt.2018.00099

**Published:** 2018-09-05

**Authors:** Daniele Agostinelli, François Alouges, Antonio DeSimone

**Affiliations:** ^1^International School for Advanced Studies (SISSA), Trieste, Italy; ^2^Centre de Mathématiques Appliquées, École Polytéchnique, Université Paris-Saclay, Paris, France; ^3^The BioRobotics Institute, Sant'Anna School for Advanced Studies, Pisa, Italy

**Keywords:** crawling motility, lumbricus terrestris, peristalsis, self-propulsion, metameric robots, biomimetic robots, soft robotics, optimization

## Abstract

*Peristalsis*, i.e., a motion pattern arising from the propagation of muscle contraction and expansion waves along the body, is a common locomotion strategy for limbless animals. Mimicking peristalsis in bio-inspired robots has attracted considerable attention in the literature. It has recently been observed that maximal velocity in a metameric earthworm-like robot is achieved by actuating the segments using a “phase coordination” principle. This paper shows that, in fact, peristalsis (which requires not only phase coordination, but also that all segments oscillate at same frequency and amplitude) emerges from optimization principles. More precisely, basing our analysis on the assumption of small deformations, we show that peristaltic waves provide the optimal actuation solution in the ideal case of a periodic infinite system, and that this is approximately true, modulo edge effects, for the real, finite length system. Therefore, this paper confirms the effectiveness of mimicking peristalsis in bio-inspired robots, at least in the small-deformation regime. Further research will be required to test the effectiveness of this strategy if large deformations are allowed.

## 1. Introduction

The study of self-propelled locomotors exploiting friction-induced traction as a result of body shape changes, is gaining attention because of the variety of physical systems which take advantage of such a locomotion strategy. One motivation is the desire to understand biological phenomena, such as cell migration on or within solid substrates, matrices, and tissues (Alberts et al., 2002). Another motivation is the attempt to replicate these mechanisms in robotics with the idea that biomimetic constructs may outperform traditional ones when confronted with unstructured and unpredictable environments.

In particular, robotic locomotion research has recently considered crawling and burrowing animals (e.g., earthworms, snakes, and caterpillars), whence an increasing number of research projects on bio-inspired metameric (soft) robots (Menciassi et al., 2006; Wang et al., 2009; Boxerbaum et al., 2012; Daltorio et al., 2013; Fang et al., 2015; Nemitz et al., 2016; Umedachi et al., 2016; Ge et al., 2017). As a matter of fact, many species such as earthworms, caterpillars, sea cucumbers and snails move using peristalsis which is a locomotion mechanism consisting of a series of wave-like muscle relaxation and contraction which propagate along the body (Quillin, 1999). One of the most studied biological species is *Lumbricus terrestris* (commonly known as *nightcrawler*) which is a kind of earthworm which uses peristalsis both for surface crawling and for burrowing. Each of its *metameres* (body segments) is endowed with longitudinal and circular muscles and can regulate frictional forces thanks to microscopic bristles called *setae* (Quillin, 1999). Understanding how relatively simple organisms are able to attain peristalsis and to which extent coordination is regulated by either the nervous system or spontaneous reflexes, are questions addressed by researchers for about a century and are still drawing attention (Garrey and Moore, 1915; Gray and Lissmann, 1938; Gardner, 1976; Quillin, 1999).

In the field of robotics, peristalsis has been mostly mimicked by *a priori* assignment of “gaits” defined by a few scalar parameters. Optimization of locomotion performances with respect to variations of these scalar parameters has been studied. Fang et al. (2015) consider harmonic deformations with a single, fixed, (time) frequency and amplitude, and determine the phase patterns of actuation maximizing the average velocity. Optimization leads to phase coordination, in the form of a pattern which is close to the *identical-phase-difference* (IPD) pattern corresponding to peristalsis. However, no rigorous proof of the connection between peristaltic waves and optimal actuation is given and, more importantly, the basic hypothesis of harmonic oscillations with a single fixed time frequency and amplitude is taken as an a priori assumption.

This paper aims to provide a deeper understanding of harmonic oscillations and peristalsis as result of an optimization problem rather than an a priori hypothesis. Indeed, we prove that - in the regime of small deformations - peristalsis is a symmetry property of the solution to an optimization problem. Symmetry of the solution comes from symmetry properties of operators in the equations governing the optimization problem, which are, in turn, the signature of geometric symmetries of the physical system.

The rest of the paper is organized in three main sections: material and methods, results and discussion. The first one, inspired by nightcrawlers' retractable *setae*, introduces a velocity-force law which is able to describe, for limit values of a single scalar parameter, the linear case (Newtonian) as well as the case of “free slip–perfect grip.” This model is presented in both continuous and discrete version and in the latter case we address some related optimal control problems. The second section illustrates the behavior of the continuous model by means of two examples and shows how peristalsis emerges, up to edge-effects, from optimization problems in the discrete framework. The last section presents a comparison with the work presented by Fang et al. (2015), states the main conclusions and provides directions for the future.

## 2. Materials and methods

### 2.1. Continuous self-propelled 1D crawlers

#### Model description and kinematics

**Figure 1:**
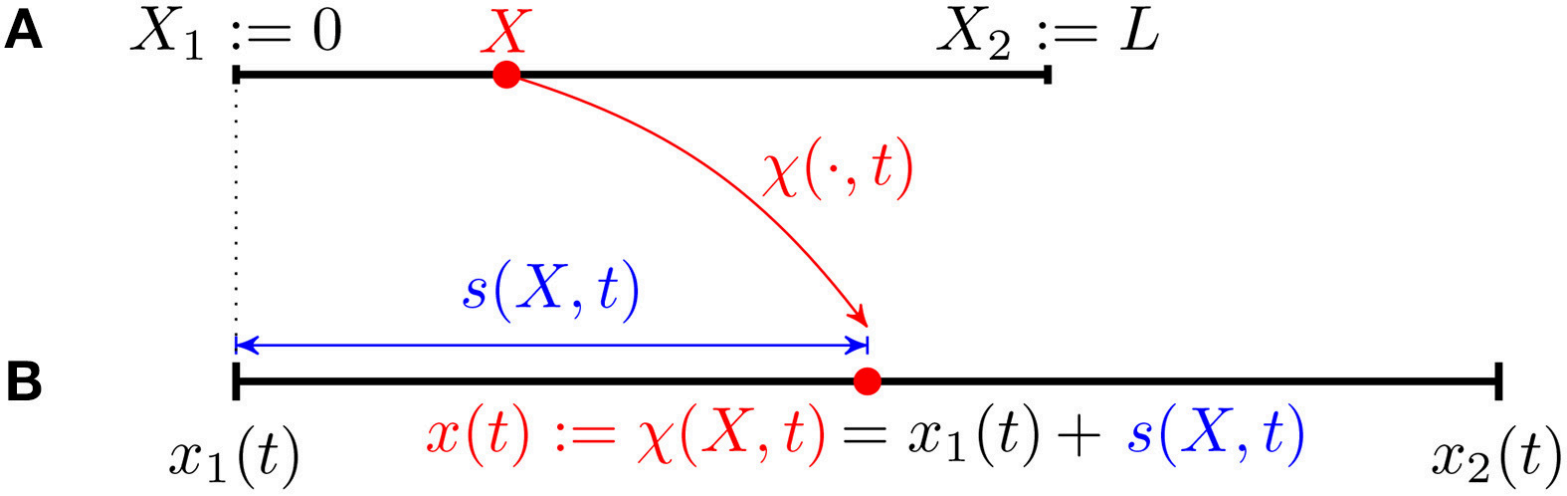
Kinematics of a continuous 1D crawler: reference **(A)** and current **(B)** configurations.

Consider a 1D crawler moving along a straight line and assume its reference configuration is the segment

$$\left[X_{1}:=0,X_{2}:=L\right],$$

cf. Figure 1. We use the same formalism introduced by DeSimone and Tatone (2012) and DeSimone et al. (2013) so that *X* is the coordinate in the reference configuration while *x*(*t*) denotes the coordinate along the crawler's body in the current configuration (at time *t*). In particular *x*(*t*) is the image of *X* through the current transformation χ(·, *t*) which can be written in terms of the current distance *s*(·, *t*) from the left end, i.e.,

$$x\left(t\right)=\chi \left(X,t\right):=x_{1}\left(t\right)+s\left(X,t\right)\in \left[x_{1}\left(t\right),x_{2}\left(t\right)\right]$$

where *x*_1_(*t*): = χ(*X*_1_, *t*) and *x*_2_(*t*): = χ(*X*_2_, *t*). By definition, *s*(0, *t*)≡0 for all *t* and we assume that

(1) $$s^{\prime }(X,t):=\frac{\partial s(X,t)}{\partial X}>0\;\forall X,t$$

in order to guarantee the monotonicity of χ(·, *t*) at any time *t*.

In what follows a prime will denote the derivative with respect to *X* while a superscript dot will denote the derivative with respect to *t*.

We define the displacement

$$u_{a}(X,t):=\chi (X,t)-X$$

so that

$$\chi^{\prime }(X,t):=\frac{\partial \chi (X,t)}{\partial X}=1+u_{a^{\prime }}(X,t)=1+\epsilon (X,t)$$

where we have implicitly defined the strain

$$\epsilon (X,t):=\frac{\partial u_{a}}{\partial X},$$

in terms of which condition (1) reads

(2) ϵ(X,t)>−1 ∀X,t.

Finally, notice that, since the material (or Lagrangian) velocity is

$$\dot{\chi }(X,t)={\dot{x}}_{1}(t)+\dot{s}(X,t),$$

the spatial (or Eulerian) velocity is given by

$$v(x,t)=\dot{\chi }(X,t){|}_{X=\chi^{-1}(x,t)}.$$

#### Equations of motion

Throughout this section we deal with the motility problem, namely, given a history of strain ϵ(*X, t*), the aim is to find *x*_1_(*t*) which determines the dynamics of the one-dimensional crawler.

##### Friction laws

The force at the interface between substrate and crawler is modelled through a force-velocity relationship. In particular, we write the density per unit current length of the tangential component of the friction force at time *t*, *f*(*x, t*), as a function of the Eulerian velocity *v*(*x, t*).

Several models for the resistance forces are conceivable such as a

**Figure 2:**
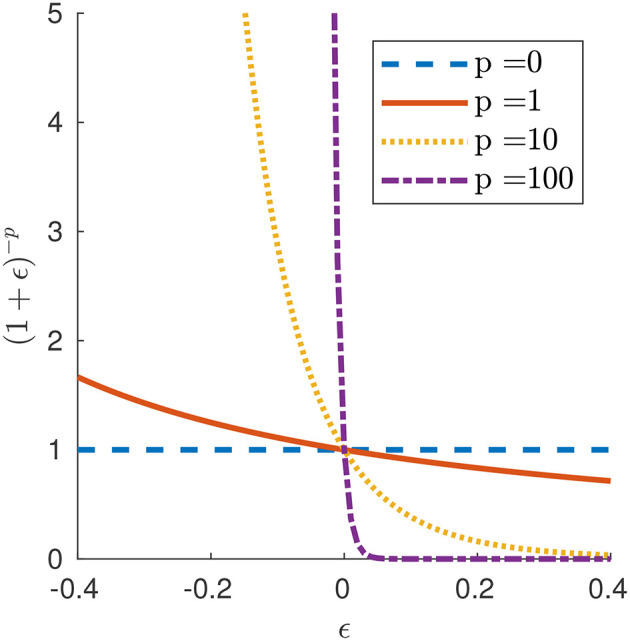
Function *g*_*p*_(ϵ) governing the friction law (4) for selected values of parameter *p*.

- Newtonian model, i.e., a linear viscous law
  (3) f(x,t):=−μv(x,t)
  where μ>0 is a friction (or viscosity) coefficient;
- or a more general “*p*-model”
  (4) $$\begin{array}{l} f(x,t):=-\mu g_{p}(\epsilon (X,t))v(x,t)\\ \text{ where }g_{p}(\epsilon ):={\left(\frac{1}{1+\epsilon }\right)}^{p} \end{array}$$
  for *p*∈[0, +∞). Parameter *p* in our force law (4) allows us to investigate different types of frictional behaviors. For *p* = 0, we obtain a force per unit current length that is a linear function of velocity alone, which reduces to the Newtonian model (3). For *p*>0, we obtain a friction law that is sensitive to the state of elongation of the segment, with force per unit length higher or lower than that of the Newtonian case depending on whether the element is contracted (λ < 1 or ϵ < 0) or extended (λ>1 or ϵ>0). In the limit *p* → ∞, this produces an idealized model for friction in which no force opposes slip when the segment is extended (free slip), while the segment can withstand any tangential force without sliding (perfect grip) when it is contracted. We call this idealized model “free slip–perfect grip.” Figure 2 displays the graphs of *g*_*p*_(ϵ) around ϵ = 0 for different values of *p*. In fact, our model is a continuous analog of the discrete model proposed by Fang et al. (2015) to mimic the behavior of earthworms' setae, which protrude when the body is axially contracted, resulting in an increment of the resistance (Edwards et al., 2018).

In what follows, we will use the *p*-model (4).

##### Force balance

The total friction is obtained by integrating the force per unit current length on the whole current domain, i.e.,

$$F_{f}(t)=\int_{x_{1}}^{x_{2}}f(x,t)dx=\int_{0}^{L}f_{ref}(X,t)dX$$

where

$$f_{ref}(X,t):=f\left(x_{1}(t)+s(X,t),t\right)s^{\prime }(X,t).$$

Then, by neglecting inertia, the force balance yields

$$\begin{array}{l} 0=F_{f}(t)+F_{e}(t)\\ =-\mu \int_{0}^{L}{\left(\frac{1}{1+\epsilon (X,t)}\right)}^{p}\left({\dot{x}}_{1}(t)+\dot{s}(X,t)\right)s^{\prime }(X,t)dX+F_{e}(t)\\ =\left[-\mu \int_{0}^{L}{\left(\frac{1}{1+\epsilon (X,t)}\right)}^{p}s^{\prime }(X,t)dX\right]{\dot{x}}_{1}\\ \;-\mu \int_{0}^{L}{\left(\frac{1}{1+\epsilon (X,t)}\right)}^{p}\dot{s}(X,t)s^{\prime }(X,t)dX+F_{e}(t)\\ =\left[-\mu \int_{0}^{L}{\left(1+\epsilon (X,t)\right)}^{1-p}dX\right]{\dot{x}}_{1}\\ \;-\mu \int_{0}^{L}{\left(1+\epsilon (X,t)\right)}^{1-p}\dot{s}(X,t)dX+F_{e}(t). \end{array}$$

The square bracket multiplying ẋ_1_(*t*) in the formula above is the drag for rigid motion at unit speed and fixed shape ϵ(*X, t*), while *F*_*e*_(*t*) is an external force which, for instance, can take into account the gravity force acting on a crawler on an inclined plane. Solving for ẋ_1_(*t*), we obtain

(5) $$\begin{array}{l} {\dot{x}}_{1}(t)=-\frac{\int_{0}^{L}{\left(1+\epsilon (X,t)\right)}^{1-p}\dot{s}(X,t)dX}{\int_{0}^{L}{\left(1+\epsilon (X,t)\right)}^{1-p}dX}\\ \;+\frac{F_{e}(t)}{\mu \int_{0}^{L}{\left(1+\epsilon (X,t)\right)}^{1-p}dX} \end{array}$$

which, in the case of zero external forces, is independent of μ.

Notice that, once the initial position *x*_1_(0) the strain ϵ(*X, t*) and the external force *F*_*e*_(*t*) are provided, the whole dynamics *x*_1_(*t*) can be determined by integrating (5). Indeed, since

(6) $$\begin{array}{l} s(X,t)=s(0,t)+\int_{0}^{X}s^{\prime }(Y,t)dY\\ \;=\int_{0}^{X}\epsilon (Y,t)dY+X \end{array}$$

one has

(7) $$\dot{s}(X,t)=\int_{0}^{X}\dot{\epsilon }(Y,t)dY$$

and hence the right hand side of (5) is known once ϵ(*X, t*) is specified.

### 2.2. Discrete self-propelled 1D crawlers

We now move to a discrete model, directly inspired by studies on annelid worms (Quillin, 1999) and metameric robots (Menciassi et al., 2006; Daltorio et al., 2013; Fang et al., 2015).

#### Model description and kinematics

**Figure 3:**
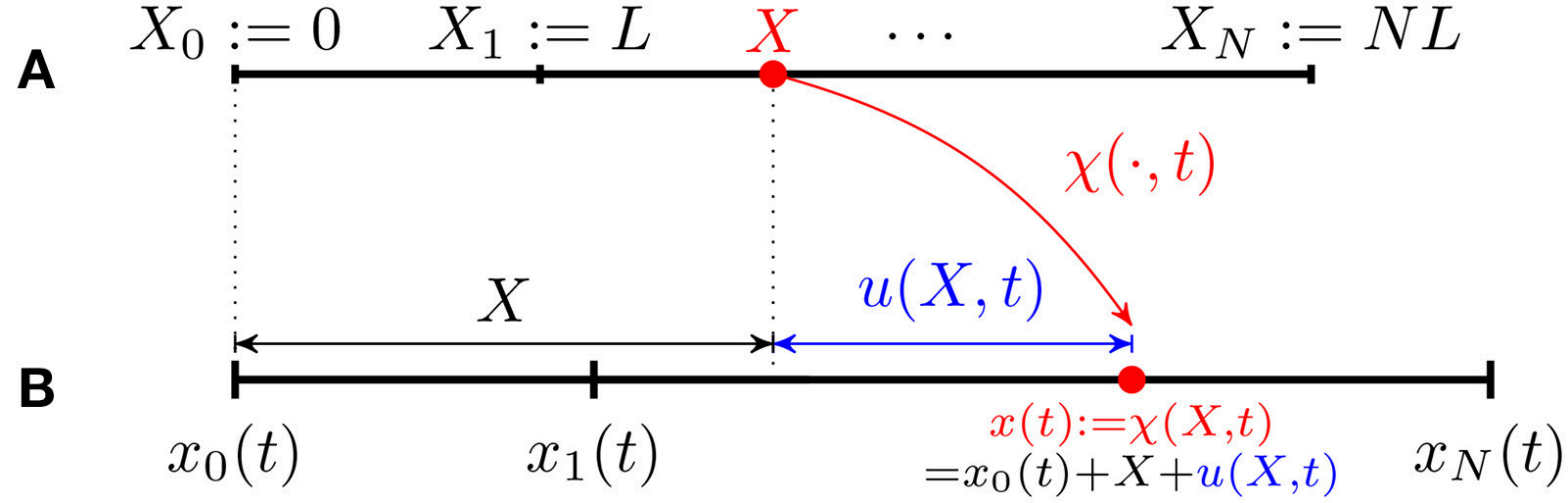
Kinematics of a discrete 1D crawler consisting of *N* identical segments of reference length L. **(A)** Reference configuration. **(B)** Current configuration.

We model the crawler's body as made up of *N* segments of same length *L* in the reference configuration (cf. Figure 3)

$$\left(X_{n-1}:=(n-1)L,\;X_{n}:=nL\right)$$

for *n* = 1, …, *N*. Let *x*_0_(*t*) denote the current position of the left edge; any point *X* in the reference domain is mapped to a point *x*(*t*) in the current domain through the map

$$x(t):=\chi (X,t)=x_{0}(t)+s(X,t)=X+u_{a}(X,t)$$

whence the definition of strain

(8) $$\epsilon (X,t):=u_{a^{\prime }}(X,t)=s^{\prime }(X,t)-1\;.$$

Furthermore, we assume that each segment can be contracted or expanded according to a constant stretch so that the overall strain results to be a piecewise constant function of *X* (at any fixed time *t*), i.e.,

(9) $$\epsilon (X,t):=\{\begin{array}{ll} \begin{array}{l} \epsilon_{1}(t)\\ \; \end{array} & \begin{array}{l} \text{if }X\in \left[X_{0},X_{1}\right)\\ \;(\text{segment 1}) \end{array}\\ \vdots & \;\\ \begin{array}{l} \epsilon_{N}(t)\\ \; \end{array} & \begin{array}{l} \text{if }X\in \left(X_{N-1},X_{N}\right]\\ \left(\text{segment N}\right) \end{array} \end{array}$$

Consequently its time-derivative, $\overset{{}^{\circ}}{\epsilon }\left(\cdot ,t\right)$, is piecewise constant and hence, from (7), ṡ(·, *t*) is piecewise affine, i.e.,

(10) $$\dot{s}(X,t)=L\sum_{j=1}^{n-1}{\dot{\epsilon }}_{j}(t)+\left[X-(n-1)L\right]{\dot{\epsilon }}_{n}(t)$$

for *X*∈[*X*_*n*−1_, *X*_*n*_]. Note that in this new framework the monotonicity condition (2) reads

$$\epsilon_{n}(t)>-1\text{ for all }t\text{ and for }n=1,\ldots ,N$$

which is the only constraint for an admissible history of strains, the datum of our motility problem.

#### Equations of motion

Analogously to the previous case, the force balance yields

$$\begin{array}{l} 0=\left[-\mu \int_{0}^{NL}{\left(1+\epsilon (X,t)\right)}^{1-p}dX\right]{\dot{x}}_{0}(t)\\ \;-\mu \int_{0}^{NL}{\left(1+\epsilon (X,t)\right)}^{1-p}\dot{s}(X,t)dX+F_{e}(t) \end{array}$$

where, in view of (9),

$$\int_{0}^{NL}{\left(1+\epsilon (X,t)\right)}^{1-p}dX=L\sum_{j=1}^{N}{\left(1+\epsilon_{j}\right)}^{1-p}$$

and, in view of (10),

$$\begin{array}{l} \int_{0}^{NL}{\left(1+\epsilon (X,t)\right)}^{1-p}\dot{s}(X,t)dX\\ \;=\sum_{n=1}^{N}{\left(1+\epsilon_{n}\right)}^{1-p}\cdot \int_{(n-1)L}^{nL}\left[\text{​}\left(X-(n-1)L\right){\dot{\epsilon }}_{n}(t)+L\sum_{k=1}^{n-1}{\dot{\epsilon }}_{k}(t)\text{​}\right]dX\\ \;=\frac{L^{2}}{2}\sum_{n=1}^{N}{\left(1+\epsilon_{n}\right)}^{1-p}\left[{\dot{\epsilon }}_{n}(t)+2\sum_{k=1}^{n-1}{\dot{\epsilon }}_{k}(t)\right]\\ \;=\frac{L^{2}}{2}\sum_{j=1}^{N}\left[{\left(1+\epsilon_{j}\right)}^{1-p}+2\sum_{m=j+1}^{N}{\left(1+\epsilon_{m}\right)}^{1-p}\right]{\dot{\epsilon }}_{j}(t). \end{array}$$

Solving for ẋ_0_(*t*) we obtain

(11) $$\begin{array}{l} {\dot{x}}_{0}(t)={\left[\sum_{j=1}^{N}{\left(1+\epsilon_{j}\right)}^{1-p}\right]}^{-1}\{\text{​}\frac{F_{e}(t)}{\mu L}\\ \;-\frac{L}{2}\sum_{j=1}^{N}\left[\text{​​}{\left(1+\epsilon_{j}\right)}^{1-p}+2\sum_{m=j+1}^{N}{\left(1+\epsilon_{m}\right)}^{1-p}\text{​​}\right]\text{​​}{\dot{\epsilon }}_{j}(t)\text{​}\} \end{array}$$

which can be rewritten in the following vectorial form

$$\begin{array}{l} {\dot{x}}_{0}(t)=\sum_{n=1}^{N}v_{n}(\epsilon (t)){\dot{\epsilon }}_{n}(t)+q(\epsilon (t))F_{e}(t)\\ \;=v(\epsilon (t))\cdot \dot{\epsilon }(t)+q(\epsilon (t))F_{e}(t) \end{array}$$

where

$$\begin{array}{l} v_{n}(\epsilon )=-\frac{L}{2}\frac{{\left(1+\epsilon_{n}\right)}^{1-p}+2\sum_{m=n+1}^{N}{\left(1+\epsilon_{m}\right)}^{1-p}}{\sum_{j=1}^{N}{\left(1+\epsilon_{j}\right)}^{1-p}},\\ q(\epsilon (t))={\left[\mu L\sum_{j=1}^{N}{\left(1+\epsilon_{j}(t)\right)}^{1-p}\right]}^{-1},\\ v(\epsilon )=\left[\begin{array}{c} v_{1}(\epsilon )\\ \vdots \\ v_{N}(\epsilon ) \end{array}\right]\text{ and }\epsilon (t)=\left[\begin{array}{c} \epsilon_{1}(t)\\ \vdots \\ \epsilon_{N}(t) \end{array}\right]. \end{array}$$

Equation (11) fully describes the dynamics once *x*_0_(0) and ϵ(*X, t*) are provided. In particular, the displacement after *T* time units is given by

(12) $$\begin{array}{l} \Delta x_{0}=x_{0}(T)-x_{0}(0)\\ \;=\int_{0}^{T}v(\epsilon (t))\cdot \dot{\epsilon }(t)dt+\int_{0}^{T}q(\epsilon (t))F_{e}(t)dt. \end{array}$$

#### Relative displacement

We can rewrite everything in terms of a displacement relative to *x*_0_(*t*), defined as

(13) $$\begin{array}{l} u(X,t):=u_{a}(X,t)-x_{0}(t)\\ \;=s(X,t)-X\\ \;=\int_{0}^{X}\epsilon (Y,t)dY \end{array}$$

in order to describe the displacement in a coordinate system which is “co-moving” with the left end *x*_0_(*t*).

In the discrete framework, the relative displacement turns out to be a piecewise affine function of *X* (at any fixed time *t*): if *X*∈[*X*_*n*−1_, *X*_*n*_],

(14) $$u(X,t)=L\sum_{j=1}^{n-1}\epsilon_{j}(t)+[X-(n-1)L]\epsilon_{n}(t).$$

Setting

$$u_{n}(t):=u(X_{n},t)\text{ for }n=1,\ldots ,N,$$

we have

$$\epsilon_{n}=\frac{u_{n}-u_{n-1}}{L}\text{ for }n=1,\ldots ,N,$$

where *u*_0_≡0. Equivalently

(15) ϵ(t)=Ju(t)

where $\epsilon ={\left(\epsilon_{1},\epsilon_{2},\ldots ,\epsilon_{N}\right)}^{T}$, $\text{u}={\left(u_{1},u_{2},\ldots ,u_{N}\right)}^{T}$ and

(16) $$J:=\frac{1}{L}\left[\begin{array}{cccc} 1 & \; & \; & \;\\ -1 & 1 & \; & \;\\ \; & \ddots & \ddots & \;\\ \; & \; & -1 & 1 \end{array}\right]\;.$$

#### Optimal control problems

In this section we address the problem of maximizing the net displacement Δ*x*_0_ among periodic shape changes ϵ(*X, t*) with the same given energy cost.

We now describe the optimization problems with quadratic energy in the non-linear case first, and then in the small-deformation regime, for which general results can be established. We assume *F*_*e*_≡0.

##### Feasible region

We assume that the shape function **ϵ**(*t*) is a *C*^2^ function defined from ℝ to ℝ^*N*^.

In addition, we require **ϵ**(·) to be a time-periodic function. Finally we restrict our search to shape functions with a given cost per period, i.e., $E\left[\epsilon ,\overset{{}^{\circ}}{\epsilon }\right]=c$, where the energy functional is assumed to be of the following quadratic form (in both **ϵ** and $\overset{{}^{\circ}}{\epsilon }$)

(17) $$E[\epsilon ,\dot{\epsilon }]:=\int_{0}^{T}A\epsilon \cdot \epsilon dt+\int_{0}^{T}B\dot{\epsilon }\cdot \dot{\epsilon }dt$$

where 𝔸 and 𝔹 are symmetric and positive definite *N*-dimensional matrices. Overall, the feasible region is

$$S:=\{\epsilon \in C^{2}\left(\mathbb{R},{\mathbb{R}}^{N}\right)\;|\;\epsilon (0)=\epsilon (T)\wedge \;|E[\epsilon ,\dot{\epsilon }]=c\}.$$

##### Optimization problem

The general (non-linear) optimization problem is

(18) $$\underset{\epsilon \in S}{\max}\;\Delta x_{0}\left[\epsilon \right]:=\int_{0}^{T}v(\epsilon (t))\cdot \dot{\epsilon }(t)dt$$

which is an isoperimetric problem (e.g., Van Brunt, 2004) involving *N* dependent variables ϵ_*n*_. The corresponding Euler-Lagrange equations lead to a second order non-linear system of ODEs, i.e., for *n* = 1, …, *N*,

(19) $$\frac{d}{dt}\frac{\partial ℱ}{\partial {\dot{\epsilon }}_{n}}(t,\epsilon ,\dot{\epsilon })-\frac{\partial ℱ}{\partial \epsilon_{n}}(t,\epsilon ,\dot{\epsilon })=0$$

where $F\left(t,\epsilon ,\overset{{}^{\circ}}{\epsilon }\right):=\text{v}\left(\epsilon \right)\cdot \overset{{}^{\circ}}{\epsilon }-\lambda \left(A\epsilon \cdot \epsilon +B\overset{{}^{\circ}}{\epsilon }\cdot \overset{{}^{\circ}}{\epsilon }\right)$ (here λ denotes a Lagrange multiplier).

##### The small-deformation regime

We can focus on the *small-deformation regime* by expanding the objective function at the leading orders (about **ϵ** = **0**), i.e.,

$$\begin{array}{l} \Delta x_{0}=\int_{0}^{T}v(\epsilon )\cdot \dot{\epsilon }dt\\ \;=\int_{0}^{T}\left(v(0)+v_{\epsilon }(0)\epsilon +o(\epsilon )\right)\cdot \dot{\epsilon }\;dt\\ \;≃v(0)\cdot \left(\epsilon (T)-\epsilon (0)\right)+\int_{0}^{T}v_{\epsilon }(0)\epsilon \cdot \dot{\epsilon }\;dt\\ \;=\int_{0}^{T}v_{\epsilon }(0)\epsilon \cdot \dot{\epsilon }\;dt \end{array}$$

and, integrating by parts,

$$\begin{array}{l} \int_{0}^{T}v_{\epsilon }(0)\epsilon \cdot \dot{\epsilon }\;dt={\left[v_{\epsilon }(0)\epsilon \cdot \epsilon \right]}_{0}^{T}-\int_{0}^{T}v_{\epsilon }(0)\dot{\epsilon }\cdot \epsilon \;dt\\ \;=\int_{0}^{T}-\dot{\epsilon }\cdot v_{\epsilon }^{T}(0)\epsilon \;dt \end{array}$$

whence

$$\begin{array}{l} \Delta x_{0}≃\frac{1}{2}\left[\int_{0}^{T}v_{\epsilon }(0)\epsilon \cdot \dot{\epsilon }dt+\int_{0}^{T}-v_{\epsilon }^{T}(0)\epsilon \cdot \dot{\epsilon }dt\right]\\ \;=\int_{0}^{T}\left(\frac{v_{\epsilon }(0)-v_{\epsilon }^{T}(0)}{2}\right)\epsilon \cdot \dot{\epsilon }dt=:V[\epsilon ,\dot{\epsilon }]. \end{array}$$

In particular, it can be proved that the (skew-symmetric Toeplitz) matrix skw(**v**_**ϵ**_(**0**)) =:*V* depends only on *N*, *L* and *p*. Indeed,

$${\left\{V\right\}}_{ij}=\{\begin{array}{ll} L(p-1)\frac{i-j+N}{2N^{2}} & \text{if }i<j\\ 0 & \text{if }i=j\\ -L(p-1)\frac{j-i+N}{2N^{2}} & \text{if }i>j \end{array}$$

(see Appendix A in Supplementary Material).

Therefore, in the regime of small deformations, problem (18) can be replaced by the following linear problem

(20) $$\underset{\epsilon \in S}{\max}\;V[\epsilon ,\dot{\epsilon }]:=\int_{0}^{T}\dot{\epsilon }\cdot V\epsilon dt.$$

The corresponding Euler-Lagrange equations

$$\frac{d}{dt}\frac{\partial ℒ}{\partial \dot{\epsilon }}(t,\epsilon ,\dot{\epsilon })-\frac{\partial ℒ}{\partial \epsilon }(t,\epsilon ,\dot{\epsilon })=0$$

where

$$ℒ(t,\epsilon ,\dot{\epsilon }):=V\epsilon \cdot \dot{\epsilon }-\lambda \left(A\epsilon \cdot \epsilon +B\dot{\epsilon }\cdot \dot{\epsilon }\right),$$

lead to the following system of second order linear ODEs

(21) $$V\dot{\epsilon }=\lambda \left(B\ddot{\epsilon }-A\epsilon \right)\;.$$

In general, a solution to (21) might be difficult to determine due to the complexity of finding a common diagonalization of 𝔸 and 𝔹. However, following the procedure adopted by Wiezel et al. (2018), we can solve this problem when one of the two operators is null, say 𝔸≡0 (resp. 𝔹≡0), and the other one, 𝔹 (resp. 𝔸), is symmetric, positive definite and such that the eigenspaces associated with the maximum-modulus eigenvalues of ${𝔹}^{-\frac{1}{2}}𝕍{𝔹}^{-\frac{1}{2}}$ (resp. ${𝔸}^{-\frac{1}{2}}𝕍{𝔸}^{-\frac{1}{2}}$) have dimension 1. Indeed, as shown in sections 1 and 2 in Appendix B (Supplementary Material), it turns out that

- for *A* = 0 and *B* symmetric and positive definite, up to a constant, a solution of (20) must be of the form
  (22) $$\epsilon (t)=-\frac{T}{\pi }ℜ\left(\alpha ie^{\frac{2\pi i}{T}t}e\right)$$
  where α∈ℂ\{0} is a constant such that $||\alpha ||=\sqrt{\frac{c}{2T}}$ and $\text{e}={\left(e_{1},e_{2},\ldots ,e_{N}\right)}^{T}\in {\mathbb{C}}^{N}\backslash \left\{0\right\}$ is a suitable constant vector depending only on *A* and *V*.
- for 𝔸 symmetric and positive definite and 𝔹 = 0, a solution of (20) with **ϵ** of unitary time frequency must be of the form
  (23) $$\epsilon (t)=2ℜ\left(\alpha e^{\frac{2\pi i}{T}t}e\right)$$
  where α∈ℂ\{0} is a constant such that $||\alpha ||=\sqrt{\frac{c}{2T}}$ and $\text{e}={\left(e_{1},e_{2},\ldots ,e_{N}\right)}^{T}\in {\mathbb{C}}^{N}\backslash \left\{0\right\}$ is a suitable constant vector depending only on 𝔹 and 𝕍.

Both (22) and (23) have the form

$$\epsilon (t)=ℜ\left(\hat{\alpha }e^{\frac{2\pi i}{T}t}e\right)$$

i.e., they are circles in the plane (ℜ(**e**), ℑ(**e**)), regardless the number of links. Moreover, using the polar representations

$$\hat{\alpha }=\varrho_{a}e^{i\vartheta_{a}}\text{ and }e_{n}=\varrho_{n}e^{i\vartheta_{n}}\;\forall n,$$

we get, for *n* = 1, …, *N*,

(24) $$\begin{array}{l} \epsilon_{n}(t)=\varrho_{a}\varrho_{n}ℜ\left(e^{i\left(\frac{2\pi }{T}t+\vartheta_{a}+\vartheta_{n}\right)}\right)\\ \;=\varrho_{a}\varrho_{n}\sin\left(\frac{2\pi }{T}t+\vartheta_{a}+\vartheta_{n}+\frac{\pi }{2}\right), \end{array}$$

i.e., the optimal gait depends only on the 2*N*+2 parameters {_ϑ_*n*_}*n*_, {_ϱ_*n*_}*n*_, ϑ_*a*_ and ϱ_*a*_. Admittedly, since α is a constant with fixed modulus and free argument, we can always assume $\vartheta_{a}+\frac{\pi }{2}=0$, i.e.,

(25) $$\epsilon_{n}(t)=\varrho_{a}\varrho_{n}\sin\left(\frac{2\pi }{T}t+\vartheta_{n}\right),$$

thus reducing the number of parameters to 2*N*+1.

The problem for 𝔸 = 0 and 𝔹 = 𝕀_*N*_ is *essentially equivalent* to the one for 𝔸 = 𝕀_*N*_ and 𝔹 = 0 (provided that unitary time frequency of **ϵ** is prescribed). Indeed, if (25) is a solution to

$$\begin{array}{l} \;\underset{\epsilon \in S}{\max}\;V[\epsilon ,\dot{\epsilon }]\\ S:=\left\{\epsilon \in C^{2}\;|\;\epsilon (0)=\epsilon (T)\wedge \int_{0}^{T}||\epsilon |{|}_{{\mathbb{R}}^{N}}^{2}dt=1\right\} \end{array}$$

then it is a solution also to

$$\begin{array}{l} \;\max_{\epsilon \in S}\;V[\epsilon ,\dot{\epsilon }]\\ S:=\{\epsilon \in C^{2}\;|\;\epsilon (0)=\epsilon (T)\int_{0}^{T}\wedge \;\int_{0}^{T}||\dot{\epsilon }|{|}_{{\mathbb{R}}^{N}}^{2}dt={\left(\frac{2\pi }{T}\right)}^{2}\} \end{array}$$

and vice versa.

In general the two problems, 𝔸 = 0 with 𝔹 symmetric positive definite and 𝔹 = 0 with 𝔸 symmetric positive definite, are not equivalent. In fact, constraining the norm induced by one operator does not determine the norm induced by the other one, but only provides a bound. Indeed, denoting by λ_*min*_(·) and λ_*max*_(·) the minimum and maximum eigenvalue respectively, observe that, for **ϵ**(*t*) like (25),

$$\begin{array}{l} \int_{0}^{T}A\epsilon \cdot \epsilon dt\geq \lambda_{min}(A)\int_{0}^{T}\epsilon \cdot \epsilon dt\\ \;=\lambda_{min}(A){\left(\frac{T}{2\pi }\right)}^{2}\int_{0}^{T}\dot{\epsilon }\cdot \dot{\epsilon }dt\\ \;\geq {\left(\frac{T}{2\pi }\right)}^{2}\frac{\lambda_{min}(A)}{\lambda_{max}(B)}\int_{0}^{T}B\dot{\epsilon }\cdot \dot{\epsilon }dt \end{array}$$

and, analogously,

$$\begin{array}{l} \int_{0}^{T}B\dot{\epsilon }\cdot \dot{\epsilon }dt\geq \lambda_{min}(B)\int_{0}^{T}\dot{\epsilon }\cdot \dot{\epsilon }dt\\ \;=\lambda_{min}(B){\left(\frac{2\pi }{T}\right)}^{2}\int_{0}^{T}\epsilon \cdot \epsilon dt\\ \;\geq {\left(\frac{2\pi }{T}\right)}^{2}\frac{\lambda_{min}(B)}{\lambda_{max}(A)}\int_{0}^{T}A\epsilon \cdot \epsilon dt\;. \end{array}$$

## 3. Results

### 3.1. Continuous model: two examples of contraction waves

In this section we discuss two examples of contraction waves to illustrate the behavior of the *p*-model. We show that the parameter *p* determines the kind of motion: for *p* < 1 the motion is prograde (i.e., motion in the same direction as the one of the waves) while for *p*>1 the model reproduces an earthworm-like retrograde motion (i.e., motion in the opposite direction as the one of the waves).

For simplicity, in the following examples we neglect external forces, i.e., *F*_*e*_≡0.

#### Smooth contraction wave

Consider a smooth traveling contraction wave by prescribing the strain along the body of the crawler as

(26) $$\epsilon (X,t):=\epsilon_{0}\cos\left(\frac{2\pi }{L}(X-ct)\right)$$

or equivalently, in terms of the stretch,

$$s^{\prime }(X,t)=1+\epsilon (X,t)=1+\epsilon_{0}\cos\left(\frac{2\pi }{L}(X-ct)\right)$$

where ϵ_0_ is the wave amplitude, *L* is the reference length of the crawler and *c* is a parameter which modulates time frequency and it is assumed to be strictly positive, i.e., the wave travels toward the right. By integrating over space,

$$\begin{array}{l} s(X,t)=s(0,t)+\int_{0}^{X}s^{\prime }(Y,t)dY\\ \;=\int_{0}^{X}\left[1+\epsilon_{0}\cos\left(\frac{2\pi }{L}(Y-ct)\right)\right]dY\\ \;=X+\int_{-2\pi \frac{ct}{L}}^{2\pi \frac{X-ct}{L}}\frac{L}{2\pi }\epsilon_{0}\cos(y)dy\\ \;=X+\frac{\epsilon_{0}L}{2\pi }\left[\sin\left(2\pi \left(\frac{X-ct}{L}\right)\right)+\sin\left(2\pi \frac{ct}{L}\right)\right] \end{array}$$

and, by differentiating with respect to time,

$$\dot{s}(X,t)=c\epsilon_{0}\left[\cos\left(2\pi \frac{ct}{L}\right)-\cos\left(2\pi \frac{\left(X-ct\right)}{L}\right)\right].$$

Finally, in view of (5),

$$\begin{array}{l} {\dot{x}}_{1}(t)=-\epsilon_{0}c\cos\left(\frac{2\pi ct}{L}\right)\\ \;+c\frac{\int_{0}^{L}\epsilon (X,t){\left(1+\epsilon (X,t)\right)}^{1-p}dX}{\int_{0}^{L}{\left(1+\epsilon (X,t)\right)}^{1-p}dX} \end{array}$$

whence

$$\begin{array}{l} x_{1}(t)=-\frac{\epsilon_{0}L}{2\pi }\sin\left(\frac{2\pi }{L}ct\right)\\ \;+c\int_{0}^{t}\frac{\int_{0}^{L}\epsilon (X,z){\left(1+\epsilon (X,z)\right)}^{1-p}dX}{\int_{0}^{L}{\left(1+\epsilon (X,z)\right)}^{1-p}dX}dz. \end{array}$$

The Newtonian case is recovered by setting *p* = 0, i.e.,

$$x_{1}(t)=-\frac{\epsilon_{0}L}{2\pi }\sin\left(\frac{2\pi }{L}ct\right)+\frac{\epsilon_{0}^{2}c}{2}t.$$

**Figure 4:**
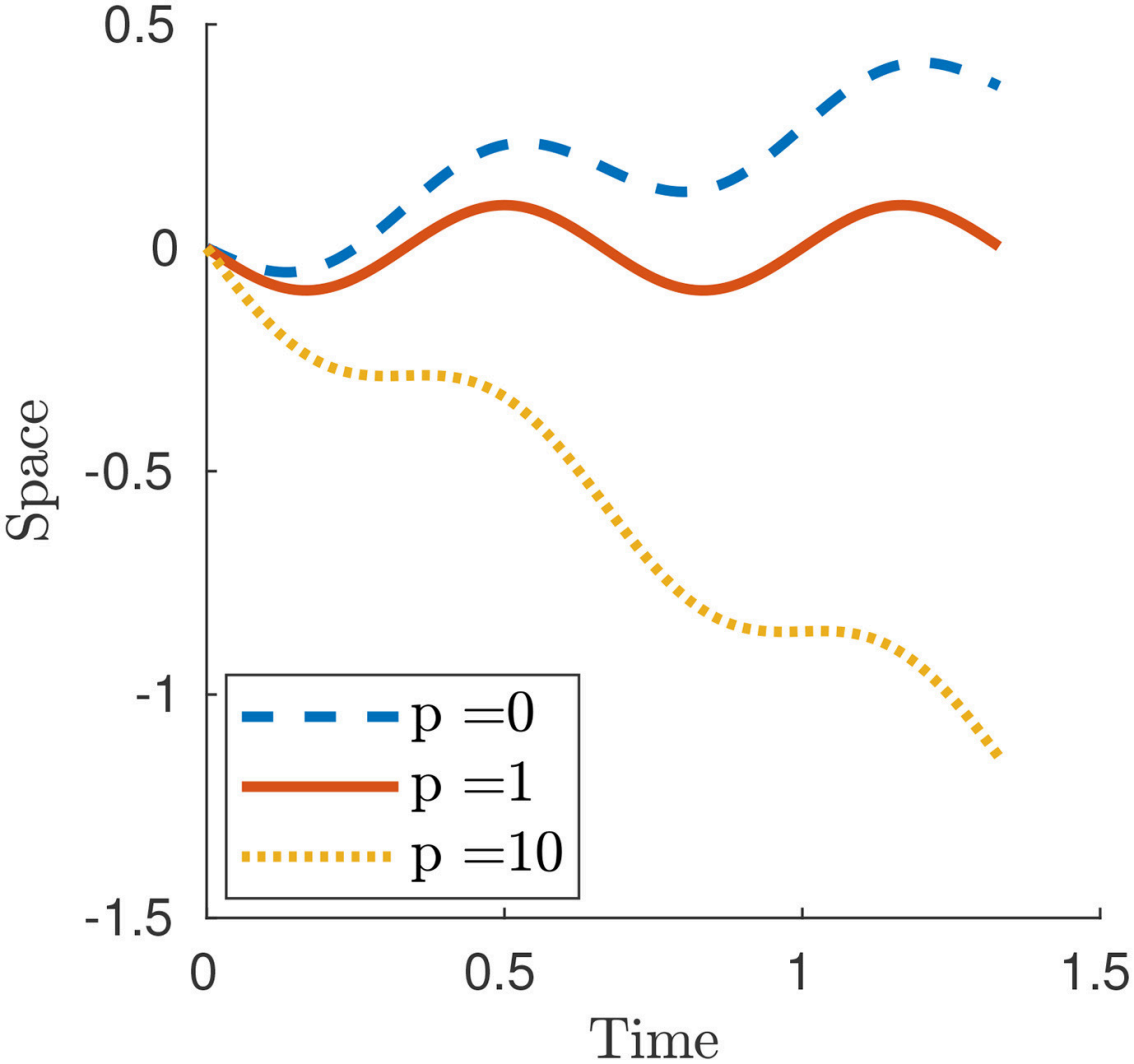
Plot of *x*_1_(*t*) for a smooth contraction wave (26) for selected values of parameter *p*. The other parameters are ϵ_0_ = 0.6, *L* = 1 and *c* = 1.5.

Figure 4 displays three numerical examples. For *p* < 1 and, in particular for *p* = 0, the case of Newtonian resistance, we always have prograde motion (i.e., motion in the same direction as the one of the waves). This is indeed observed for example in snails, although in this case the force-velocity laws that we use in this paper would not be fully adequate to capture the properties of the mucus present between the animal and the surface [non-Newtonian rheology, suction effects, see (Denny, 1980) and (DeSimone et al., 2013)]. For *p*>1 and, in particular, for the limit case *p* = ∞ describing the perfect-grip/free-slip ideal version of the modulated friction laws typical of animals with setae, the motion is retrograde (i.e., motion in the opposite direction as the one of the waves). This is the behavior typically observed for earthworms.

#### Square contraction wave

Consider the square contraction wave

(27) $$\begin{array}{l} \;\epsilon (X,t):=\epsilon_{0}(X-ct)\\ \text{where }\epsilon_{0}(x):=\{\begin{array}{l} \begin{array}{ll} \delta & \text{if }x_{\sim L}\leq \xi \\ -\delta & \text{if }x_{\sim L}>\xi \end{array} \end{array} \end{array}$$

or equivalently, in terms of the stretch,

$$\begin{array}{l} s^{\prime }(X,t)=1+\epsilon (X,t)\\ \;=\{\begin{array}{l} \begin{array}{ll} 1+\delta & \text{if }{\left(X-ct\right)}_{\sim L}\leq \xi \\ 1-\delta & \text{if}{\left(X-ct\right)}_{\sim L}>\xi \end{array} \end{array} \end{array}$$

where *L* is the reference length of the crawler, *c* is the wave speed, ξ is the measure of the interval where ϵ = δ and the subscript _~*L*_ denotes the “modulo *L*” operator (i.e., *y*_~*L*_ stands for *y*mod*L*).

By integrating the stretch over space, we get

$$\begin{array}{l} s(X,t)=s(0,t)+\int_{0}^{X}s^{\prime }(Y,t)dY\\ \;=X+\int_{0}^{X}\epsilon_{0}(Y-ct)dY \end{array}$$

whence

$$\dot{s}(X,t)=\{\begin{array}{ll} -2\delta c & \text{if }ct_{\sim L}\leq L-\xi \\ \; & \wedge \;X\in {\left[ct,ct+\xi \right]}_{\sim L}\\ 2\delta c & \text{if }ct_{\sim L}>L-\xi \\ \; & \wedge \;X\in {\left[ct+\xi -L,ct\right]}_{\sim L}\\ 0 & \text{else} \end{array}$$

Finally, in view of (5),

$${\dot{x}}_{1}(t)=\{\begin{array}{ll} A(p) & \text{if }ct_{\sim L}\leq L-\xi \\ B(p) & \text{otherwise} \end{array}$$

where

$$A(p):=\frac{2\delta c{\left(1+\delta \right)}^{1-p}\xi }{{\left(1+\delta \right)}^{1-p}\xi +{\left(1-\delta \right)}^{1-p}(L-\xi )}$$

and

$$B(p):=\frac{2\delta c{\left(1-\delta \right)}^{1-p}(\xi -L)}{{\left(1+\delta \right)}^{1-p}\xi +{\left(1-\delta \right)}^{1-p}(L-\xi )}.$$

**Figure 5:**
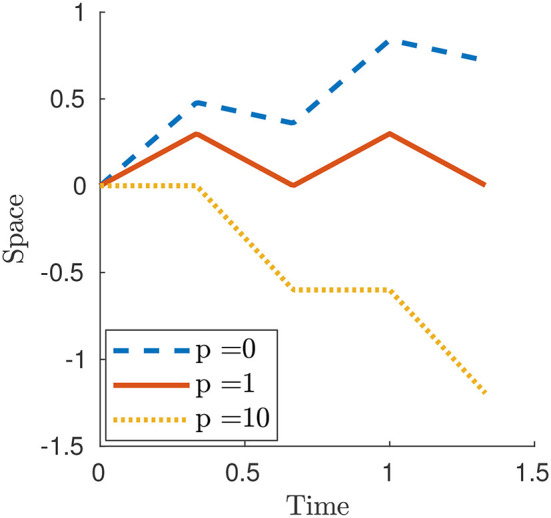
Plot of *x*_1_(t) for a square contraction wave (27) for selected values of parameter *p*. The other parameters are δ = 0.6, *L* = 1, *T* = 0.5 and *c* = 1.5.

Defining $\alpha :=\frac{L-\xi }{c}$ and $\beta :=\frac{L}{c}$, it follows that

$$\begin{array}{l} x_{1}(t)=x_{1}(0)+\int_{0}^{t}\dot{x_{1}}(z)dz\\ \;=\int_{0}^{\left\lfloor \frac{t}{\beta }\right\rfloor \beta }\dot{x_{1}}(z)dz+\int_{\left\lfloor \frac{t}{\beta }\right\rfloor \beta }^{\left(\frac{t}{\beta }\right)\beta }\dot{x_{1}}(z)dz\\ \;=\left\lfloor \frac{t}{\beta }\right\rfloor \left[\alpha A(p)+\left(\beta -\alpha \right)B(p)\right]+\\ \;\{\begin{array}{ll} \left\{\frac{t}{\beta }\right\}\beta A(p) & \text{if }\left\{\frac{t}{\beta }\right\}\beta \leq \alpha \\ \alpha A(p)+\left(\left\{\frac{t}{\beta }\right\}\beta -\alpha \right)B(p) & \text{else} \end{array} \end{array}$$

where {·} and ⌊·⌋ denote the fractional part and floor function respectively. The Newtonian case can be obtained as particular case by setting *p* = 0. Figure 5 shows three numerical examples and, as for the smooth contraction wave, the motion is prograde or retrograde whether *p* < 1 or *p*>1, respectively.

### 3.2. Discrete model: peristalsis as optimal gait

In the discrete framework, peristalsis is the result of phase coordination among the harmonic contractions of body segments, i.e., it has the form

$$\epsilon_{n}\left(t\right)=\varrho \sin\left(\frac{2\pi t}{T}+n\Delta \varphi \right)\;\text{for}n=1,\ldots ,N$$

where *T* is the period, ϱ is the amplitude and Δφ is the constant phase difference. As for the continuous case, discrete peristalsis produces prograde or retrograde motions according to the value of the parameter *p* in (4).

In this section we work out explicitly the problem of maximizing the displacement for a particular case from which peristalsis emerges, modulo an *edge-effect*.

#### Dissipation energy

Let us define an energy functional *D*:*C*^2^(ℝ, ℝ^*N*^) → ℝ as

$$D\left[\epsilon \right]:=\int_{0}^{T}d(t,\epsilon ,\dot{\epsilon })dt$$

where

$$\begin{array}{l} \;d(t,\epsilon ,\dot{\epsilon }):=d_{1}(t,\epsilon ,\dot{\epsilon })+wd_{2}(t,\dot{\epsilon }),\\ d_{1}(t,\epsilon ,\dot{\epsilon }):=\int_{0}^{NL}-\frac{1}{\mu }f_{ref}(X,t)v(\chi (X,t))dX,\\ \;d_{2}(t,\dot{\epsilon }):=\sum_{n=1}^{N}{\dot{\epsilon }}_{n}^{2}(t) \end{array}$$

i.e., the energy cost is the time integral over a period of a dissipation rate which is sum of two terms: $d_{1}\left(t,\epsilon ,\overset{{}^{\circ}}{\epsilon }\right)$ is $\frac{1}{\mu }$ times the energy expended to overcome the friction force and $d_{2}\left(t,\overset{{}^{\circ}}{\epsilon }\right)$ is the cost of control weighted by a scalar factor *w*. *D*[**ϵ**] is thus $\frac{1}{\mu }$ times the sum of the work due to the friction force plus the *L*^2^-norm of the controls suitably weighted to time the input direction.

Some calculations [see section 1 in Appendix C (Supplementary Material)] lead to

$$d_{1}(t,\epsilon ,\dot{\epsilon })=\dot{\epsilon }\cdot D(\epsilon )\dot{\epsilon }$$

where 𝔻(**ϵ**)∈ℝ^*N*×*N*^ for any **ϵ**∈(−1, +∞]^*N*^, and

$$d_{2}(t,\dot{\epsilon })=\dot{\epsilon }\cdot I_{N}\dot{\epsilon }$$

where 𝕀_*N*_ is the *N*-dimensional identity matrix. Therefore the energy functional is

(28) $$D\left[\epsilon \right]=\int_{0}^{T}\dot{\epsilon }\cdot G(\epsilon )\dot{\epsilon }dt$$

where 𝔾(**ϵ**): = 𝔻(**ϵ**)+*wI*_*N*_.

#### Non-linear optimal control problem

The non-linear optimization problem associated with energy functional (28) is

(29) $$\begin{array}{l} \;\underset{\epsilon \in S}{\max}\;\Delta x_{0}:=\int_{0}^{T}v(\epsilon )\cdot \dot{\epsilon }dt\\ S=\left\{\epsilon \in C^{2}\;|\;\epsilon (0)=\epsilon (T)\;\wedge \;D[\epsilon ]=c\right\}. \end{array}$$

The Euler-Lagrange equations lead to a second order non-linear system of ODEs, i.e., for *n* = 1, …, *N*,

(30) $$\frac{d}{dt}\frac{\partial ℱ}{\partial {\dot{\epsilon }}_{n}}(t,\epsilon ,\dot{\epsilon })-\frac{\partial ℱ}{\partial \epsilon_{n}}(t,\epsilon ,\dot{\epsilon })=0$$

where $F\left(t,\epsilon ,\overset{{}^{\circ}}{\epsilon }\right):=\text{v}\left(\epsilon \right)\cdot \overset{{}^{\circ}}{\epsilon }-\lambda \overset{{}^{\circ}}{\epsilon }\cdot G\left(\epsilon \right)\overset{{}^{\circ}}{\epsilon }$, λ is the Lagrange multiplier.

#### The small-deformation regime

In the regime of small deformations we can expand the terms of problem (29) at the leading orders about **ϵ** = **0**. As before, the net displacement per time period can be approximated by

$$\Delta x_{0}≃V[\epsilon ,\dot{\epsilon }]:=\int_{0}^{T}V\epsilon \cdot \dot{\epsilon }dt.$$

and the energy functional by

(31) $$D[\epsilon ]=\int_{0}^{T}\dot{\epsilon }\cdot G(\epsilon )\dot{\epsilon }dt≃\int_{0}^{T}\dot{\epsilon }\cdot G\dot{\epsilon }dt$$

where 𝔾: = 𝔾(**0**). Hence, in the small-deformation regime, the problem fits the form (17)-(18) for 𝔸 = 0 and 𝔹 = 𝔾. Moreover, *G* is bisymmetric (namely, symmetric about both of its diagonals) and depends only on *N*, *L* and *w*. Indeed

$${\left\{G\right\}}_{ij}=\{\begin{array}{ll} \frac{L^{3}}{4N}(2i-1)\left(2(N-j)+1\right) & \text{if }i<j\\ \frac{L^{3}}{12N}\left[4N(3i-2)-3{\left(2i-1\right)}^{2}\right]+w & \text{if }i=j\\ \frac{L^{3}}{4N}(2j-1)\left(2(N-i)+1\right) & \text{if }i>j \end{array}$$

[see section 2 in Appendix C (Supplementary Material)]. Therefore a solution must be of the form (25)

$$\epsilon_{n}^{⋆}(t)=\varrho_{a}\varrho_{n}\sin\left(\frac{2\pi }{T}t+\vartheta_{n}\right).$$

**Figure 6:**
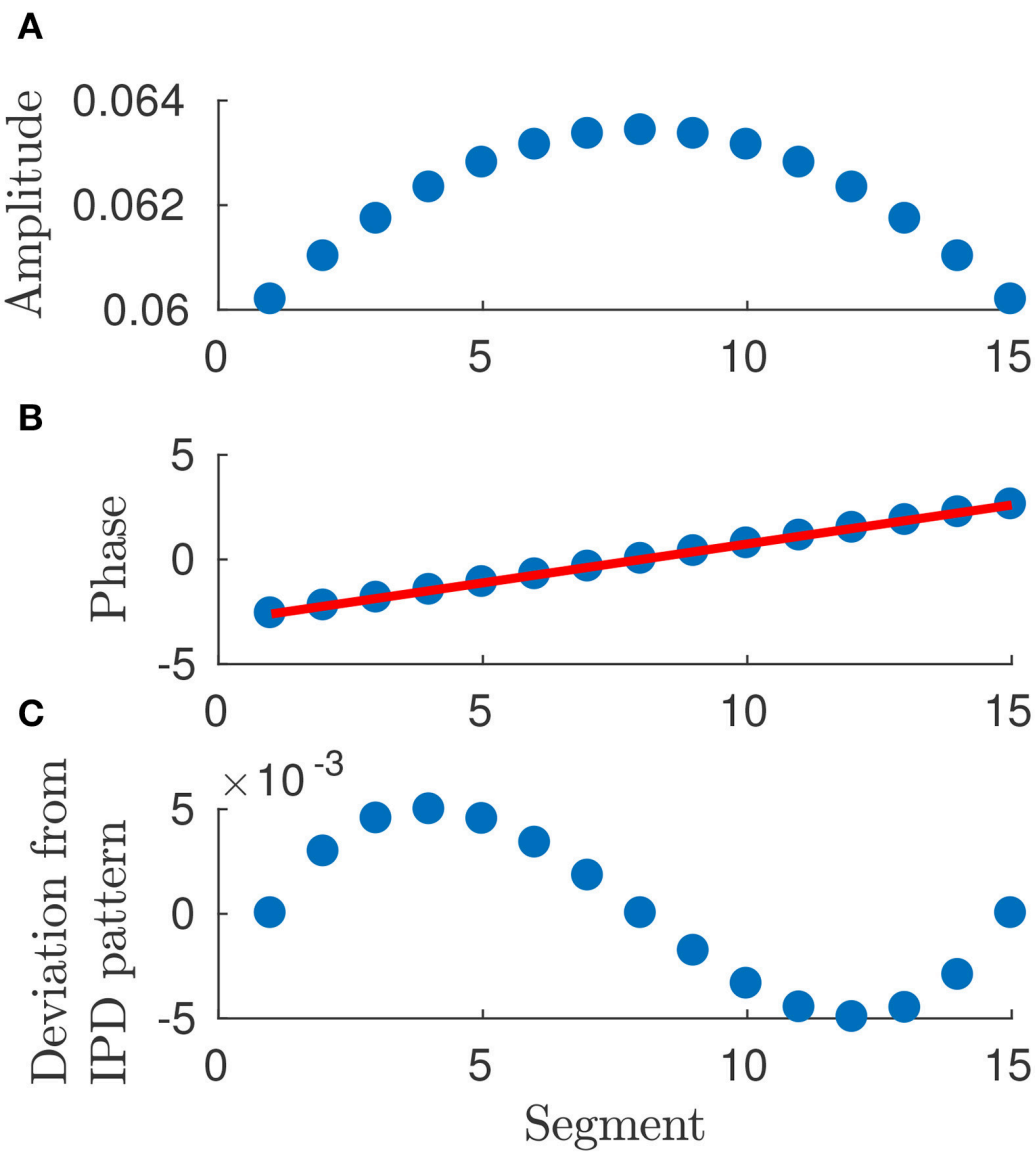
Plot of arguments and moduli of ϵ_*n*_ for *n* = 1, …, 15: amplitudes, **(A)**, approximation by a IPD (Identical Phase Difference) model, **(B)**, and relative errors, **(C)**. Parameters: *p* = 100, *w* = 1, *T* = 1 and *L* = 1.

The centrosymmetry of 𝔾 and the skew-centrosymmetry of 𝕍 imply a *reflectional symmetry* about the center [see section 3 in Appendix B (Supplementary Material)], i.e.,

- the moduli of components of **e** are symmetric about the center (cf. Figure 6), i.e.,
  $$\varrho_{N+1-n}=\varrho_{n}\;\forall \;n=1,\ldots ,N;$$
- phase differences between adjacent segments are symmetric about the center, i.e.,
  $$\vartheta_{n+1}-\vartheta_{n}=\vartheta_{N+1-n}-\vartheta_{N-n}\;\forall \;n=1,\ldots ,N$$
  so that the *N*-th phase differs from the (*N*−1)-th one by the same amount by which the second phase differs from the first one and so on (cf. Figure 6).

Equation (25) shows that the optimal gait requires a precise “phase coordination” of locomotion patterns among the segments, which is a common observation in Biology for several kinds of animals.

Numerical simulations show that the optimal solution turns out to be a discrete approximation of a traveling wave. In particular,

- the moduli of *e*_*n*_ for *n* = 1, …, *N* can be approximated by a constant average value (cf. Figure 6), i.e.,
  (32) $$\varrho_{n}≃\bar{\varrho }\text{ constant}$$
  so that each segment undergoes a harmonic deformation with a certain initial phase;
- phase differences between adjacent segments turn out to be almost constant, i.e., for a suitable φ_0_
  (33) $$\vartheta_{n}≃n\vartheta^{⋆}+\vartheta_{0}$$
  holds true for *n* = 1, …, *N* (cf. Figure 6).

**Figure 7:**
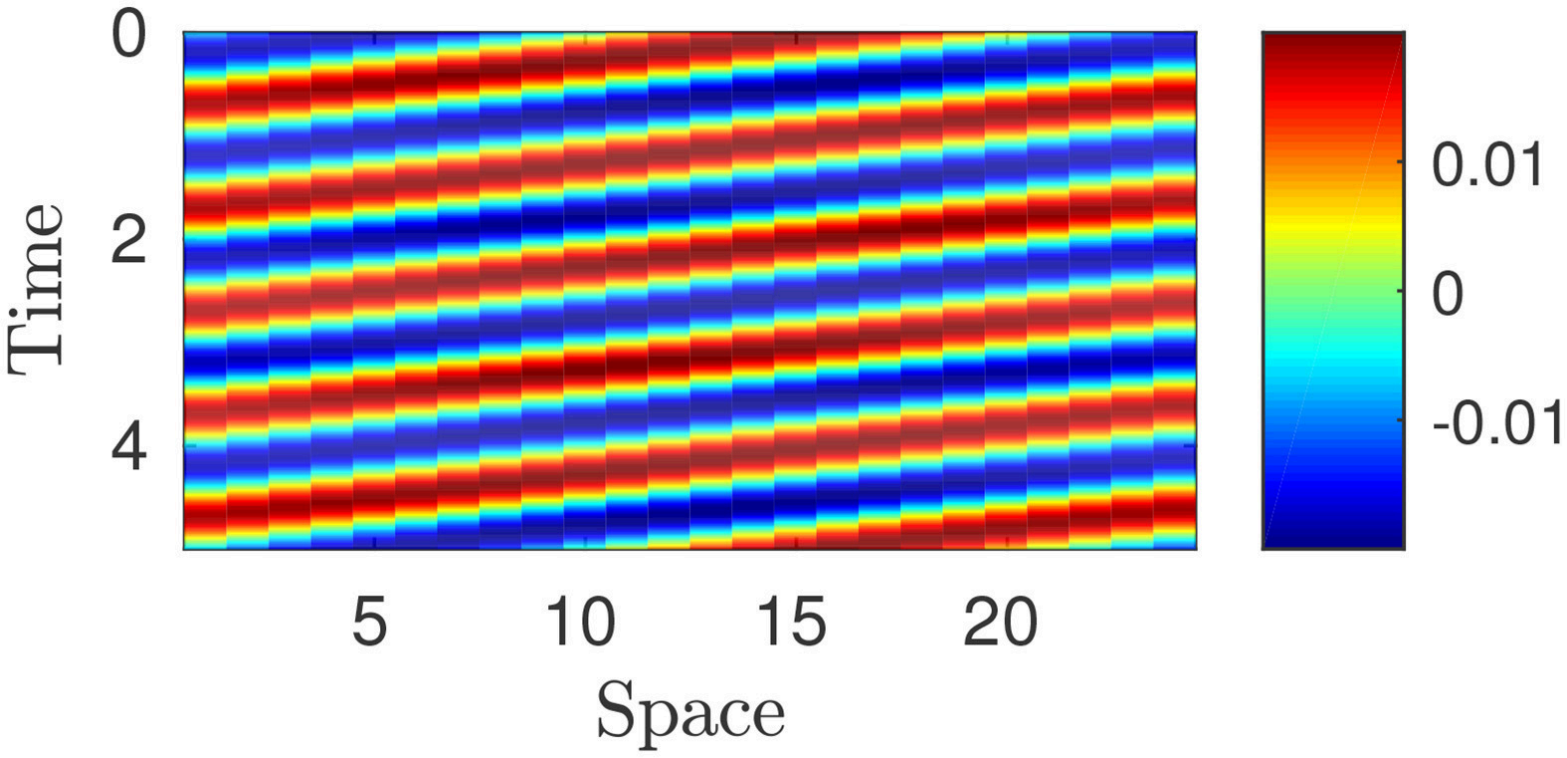
Plot of piecewise constant optimal strain $\epsilon_{n}^{⋆}\left(X,t\right)$: the value is determined by means of the color legend. Parameters: *p* = 100, *w* = 10, *T* = 1, *L* = 1; *N* = 25.

Therefore, in view of (32) and (33), the solution is a discrete approximation of a continuous traveling wave, i.e.,

$$\epsilon_{n}^{⋆}(t)=\epsilon^{⋆}(Y_{n},t)$$

where $Y_{n}=\frac{X_{n}+X_{n+1}}{2}$ is the midpoint of the *n*-th segment and

$$\begin{array}{l} \epsilon^{⋆}(X,t)=\varrho_{a}\varrho (X)\sin\left(\frac{2\pi }{T}t+\vartheta (X)\right)\\ \;≃\varrho_{a}\bar{\varrho }\sin\left(\frac{2\pi }{T}t+\vartheta^{⋆}X+\vartheta_{0}\right)\\ \;=\varrho_{a}\bar{\varrho }\sin\left(\vartheta^{⋆}\left[X-\left(-\frac{1}{\vartheta^{⋆}}\frac{2\pi }{T}\right)t\right]+\vartheta_{0}\right) \end{array}$$

whence the canonical form of traveling wave which describes peristalsis (cf. Figure 7)

(34) $$\epsilon^{⋆}(X,t)≃H(X-vt)$$

where $H\left(y\right):=\varrho_{a}\bar{\varrho }\sin\left(\vartheta^{⋆}y+\vartheta_{0}\right)$ and $v:=-\frac{1}{\vartheta^{⋆}}\frac{2\pi }{T}$.

#### The edge-effect

The symmetric structure of the optimal gait (in the small-deformation regime) arises from underlying physical symmetries which clearly stand out in the properties of the matrices 𝔾 and 𝕍. In particular, an “edge-effect” is apparent: the 1D crawler is symmetric about its geometric center and segments near the edges behave differently with respect to adjacent segments, but in the same way as their centrosymmetric counterparts.

As expected, this edge-effect vanishes when considering an “infinite” (periodic) 1D crawler because, due to the shift-invariance symmetry, each segment behaves as a “geometric center.”

**Figure 8:**
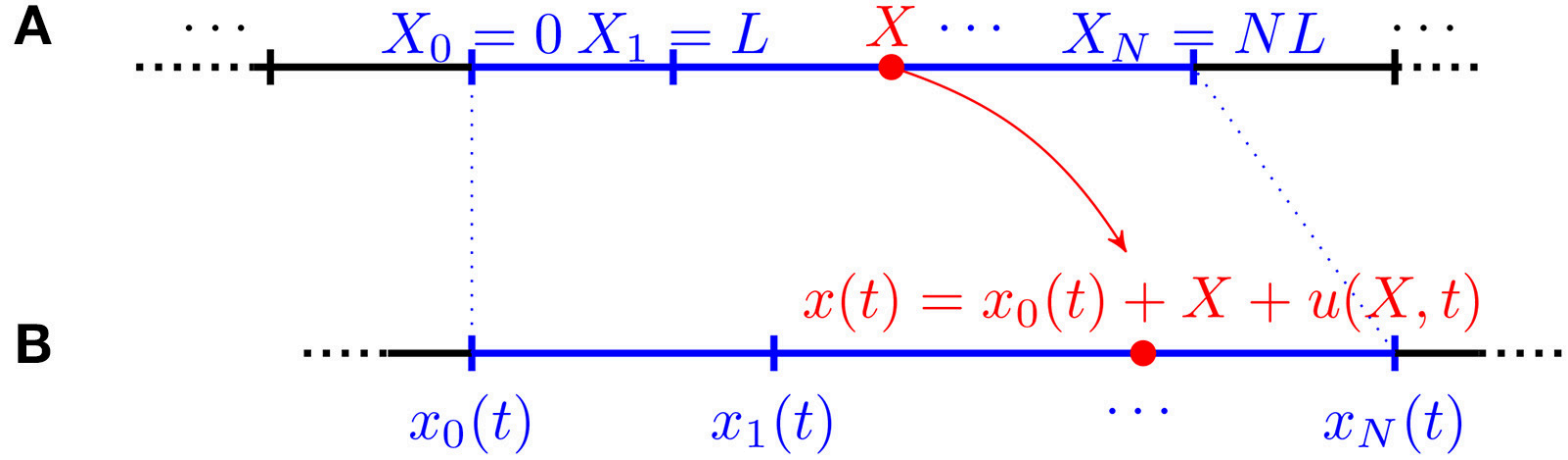
Kinematics of a discrete infinite 1D crawler consisting of identical segments of reference length *L*. **(A)** Reference configuration. **(B)** Current configuration.

To show this claim consider a 1D crawler made up of infinitely many segments and assume that it is a periodic structure of which each module consists of *N* components (cf. Figure 8).

At any time t, we already defined the relative displacement *u*(·, *t*) as the change of position of the material point *X* in the body's reference, i.e.,

$$x(t)=\chi (X,t)=x_{0}(t)+X+u(X,t).$$

The hypothesis of periodicity leads to

(35) u(X+NL,t)=u(X,t) ∀ X,t.

From (35) we obtain that the friction force is periodic and we can consider the force balance in a single module. Therefore, condition (35) reads

(36) $$\int_{0}^{NL}\epsilon (X,t)dX=0\;\forall \;t$$

and, in the discrete framework (9), this leads to

(37) $$\sum_{n=1}^{N}\epsilon_{n}(t)=0\;\forall \;t.$$

**Figure 9:**
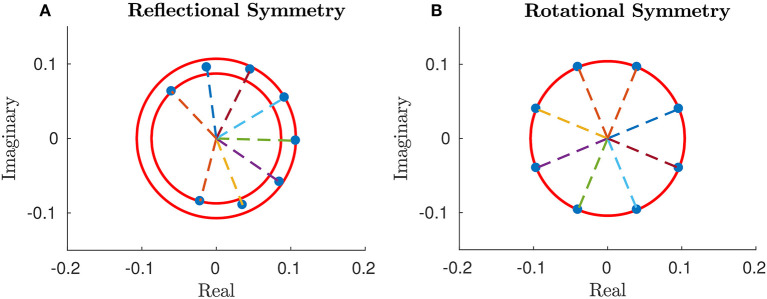
Complex components of the vector **e** in the general case, **(A)**, and in the periodic one, **(B)**, for *N* = 8 segments. Parameters: *p* = 100, *w* = 10, *T* = 1, *L* = 1.

The optimal control problem becomes

(38) $$\begin{array}{l} \;\underset{\epsilon \in S^{\prime }}{\max}\;V[\epsilon ,\dot{\epsilon }]:=\int_{0}^{T}\dot{\epsilon }\cdot V\epsilon dt\\ S^{\prime }=\{\epsilon \int_{0}^{T}\in C^{2}\left(\mathbb{R},{\mathbb{R}}^{N}\right)\;|\;\sum_{n=1}^{N}\epsilon_{n}=0\;\wedge \\ \;\epsilon (0)=\epsilon (T)\;\wedge \;\int_{0}^{T}\dot{\epsilon }\cdot G\dot{\epsilon }dt=c\} \end{array}$$

and it can be proved [see section 3 in Appendix C (Supplementary Material)] that its solutions need to be like (22), where the complex *N*-dimensional vector **e** has the form

(39) $$e=\left[\begin{array}{c} \begin{array}{c} e_{1}\\ e_{2}\\ \vdots \\ e_{n}\\ \vdots \\ e_{N} \end{array} \end{array}\right]=\left[\begin{array}{c} \begin{array}{c} e_{1} \end{array}\\ e^{i\frac{2\pi k}{N}}e_{1}\\ \vdots \\ e^{i\frac{2\pi k}{N}(n-1)}e_{1}\\ \vdots \\ e^{i\frac{2\pi k}{N}(N-1)}e_{1} \end{array}\right]$$

for some *k*∈{1, …, *N*−1} and *e*_1_∈ℂ\{0}, cf. Figure 9. In particular,

- each component of **e** has modulus ϱ: = ||*e*_1_||;
- each component can be obtained from the previous one by a rotation of $\frac{2\pi k}{N}$ or, in other words, the phase difference between two consecutive components is constant, i.e., for *n* = 1, …, *N*
  $$\arg(e_{n})=(n-1)\frac{2\pi k}{N}+\arg(e_{1})=n\vartheta^{⋆}+\vartheta_{0}$$
  where $\vartheta^{⋆}:=\frac{2\pi k}{N}$ and $\vartheta_{0}:=\arg\left(e_{1}\right)-\frac{2\pi k}{N}$;

whence the exact harmonic peristalsis.

Notice that problem (38) can be written in terms of relative displacements *u*_*n*_ through the periodic version of transformation (15), i.e.,

(40) $$\epsilon (t)=J_{per}u(t)$$

where

(41) $$J_{per}:=\frac{1}{L}\left[\begin{array}{cccc} 1 & \; & \; & -1\\ -1 & 1 & \; & \;\\ \; & \ddots & \ddots & \;\\ \; & \; & -1 & 1 \end{array}\right].$$

In particular, we get

(42) $$\begin{array}{l} \;\underset{\epsilon \in S_{u}^{⋆}}{\max}\;V[u,\dot{u}]:=\int_{0}^{T}\dot{u}\cdot V_{u}^{⋆}udt\\ S_{u}^{⋆}=\{u\in \int_{0}^{T}C^{3}\left(\mathbb{R},{\mathbb{R}}^{N}\right)\;|\;u(0)=u(T)\\ \;\wedge \;E\left[u,\dot{u}\right]:=\int_{0}^{T}\dot{u}\cdot G_{u}^{⋆}\dot{u}dt=c\} \end{array}$$

where

$$\begin{array}{l} V_{u}^{⋆}:=J_{per}^{T}VJ_{per}\\ G_{u}^{⋆}:=J_{per}^{T}GJ_{per} \end{array}$$

are *circulant* matrices (namely, Toeplitz matrices where each row vector is rotated one element to the right relative to the preceding row vector), thus reflecting the geometric symmetry of the periodic structure, namely, the *shift-invariance*.

Considering the general (i.e., non-periodic) problem in terms of relative displacements yields

$$\begin{array}{l} \;\underset{\epsilon \in S_{u}}{\max}\;V[u,\dot{u}]:=\int_{0}^{T}\dot{u}\cdot V_{u}udt\\ S_{u}=\{\int_{0}^{T}u\in C^{3}\left(\mathbb{R},{\mathbb{R}}^{N}\right)\int_{0}^{T}|\;u(0)=u(T)\\ \;\wedge \;E\left[u,\dot{u}\right]:=\int_{0}^{T}\dot{u}\cdot G_{u}\dot{u}dt=c\} \end{array}$$

where

$$\begin{array}{l} V_{u}:=J^{T}VJ\\ G_{u}:=J^{T}GJ \end{array}$$

are “*quasi-circulant*” matrices indeed

$$\begin{array}{l} V_{u}:=V_{u}^{⋆}+E_{V}\\ G_{u}:=G_{u}^{⋆}+E_{G} \end{array}$$

where 𝔼_*V*_ and 𝔼_*G*_ are null a part from the last column and the last row, i.e.,


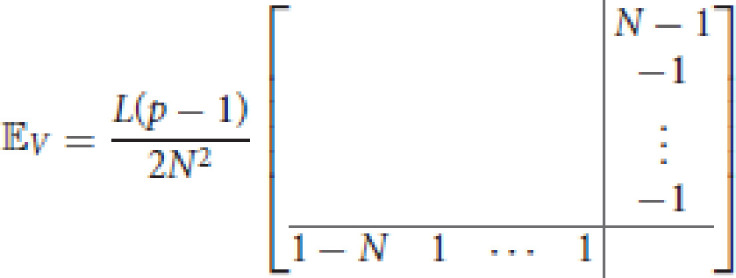


and


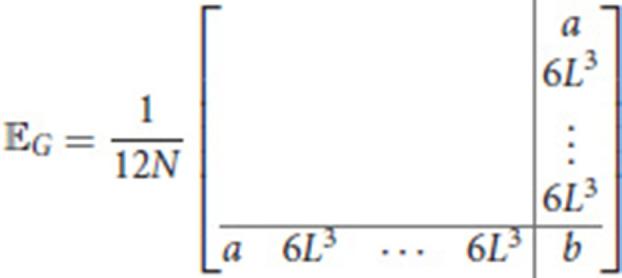


where *a* = 2*L*^3^(3−*N*)+12*Nw* and *b* = *L*^3^(9−4*N*)−12*Nw*.

#### Wavenumber

**Figure 10:**
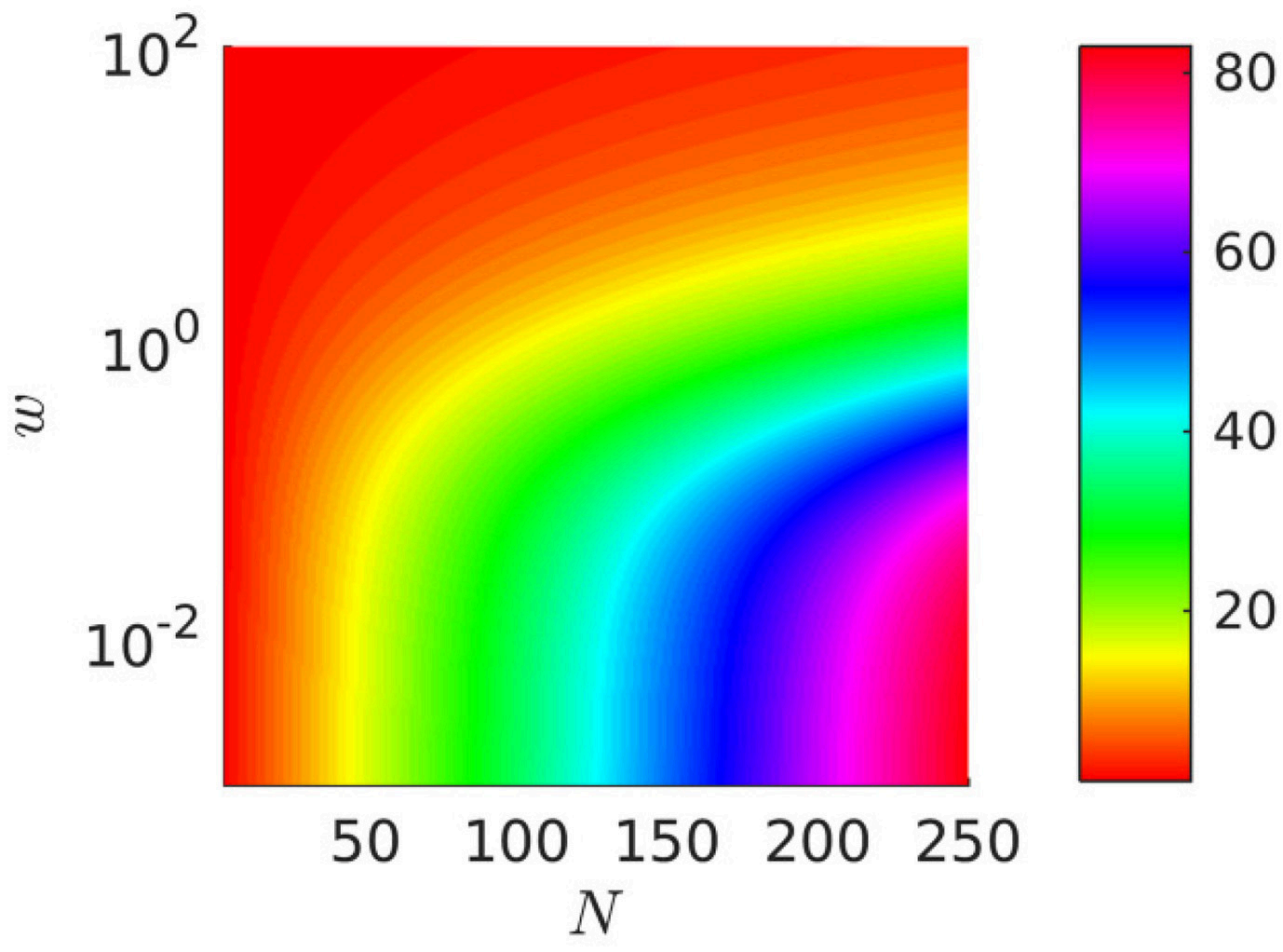
Wavenumber of optimal gaits as a function of *N* and *w*. The axis of *w*∈[0, 100] is plotted on a log-scale with base 10. The color-bar gives the wavenumber. *L* = 1 and *N*∈[3, 250].

In the “periodic case” we can study the wavenumber (that is the number of waves travelling along the body of the crawler) of the optimal gait in relation to the number of metameres *N* and to the weight *w*. We fix the dissipation

$$E[\epsilon ,\dot{\epsilon }]:=\int_{0}^{T}\left(d_{1}(t,\epsilon ,\dot{\epsilon })+wd_{2}(t,\epsilon ,\dot{\epsilon })\right)dt=\bar{c}$$

and we let *w* vary from 0 to 10^2^ for *N*∈[3, 250] (see Figure 10). As shown in Section 3 in Appendix C (Supplementary Material), the wavenumber of the optimal gait must be an integer close to the real number $\frac{N}{2\pi }\arccos\left(\frac{1}{2}\frac{6w-L^{3}}{3w+L^{3}}\right)$ and hence, for any fixed *N*, it depends on the order of magnitude of the weight *w* and

- for *w* → ∞, it tends to 1, corresponding to a single wave spanning the whole length *NL*;
- for *w* = 0, it is close to $\frac{N}{3}$, i.e., one full wave-length every three segments.

This behavior is qualitatively unaffected by the type of friction model which is adopted (i.e., by the choice of the parameter *p*).

## 4. Discussion

### 4.1. Comparison with previous studies

To put our study in perspective, we consider the discrete framework and we compare our results with the ones presented by Fang et al. (2015). Here the authors perform an optimization of the so-called “average steady-state velocity” *u*_*s*_ among harmonic shape functions having the form (in our notation)

(43) $$\epsilon_{n}(t)=a\sin\left(\frac{2\pi }{T}t+\eta_{n}\right)\text{ for }n=1,\ldots ,N$$

where $a\in \left(0,\frac{1}{L}\right)$ is the oscillation amplitude, *T* is the period and η_*n*_ is the actuation phase for the *n*-th segment (or actuator).

Since the average steady-state velocity is given by

(44) $$u_{s}=\frac{\Delta x_{0}}{T}=\frac{1}{T}\int_{0}^{T}v(\epsilon )\cdot \dot{\epsilon }dt\;,$$

the optimization problem reads

(45) $$\underset{\eta \in {\left[0,2\pi \right)}^{N}}{\max}\;u_{s}(\eta )=\frac{1}{T}\int_{0}^{T}v(\epsilon )\cdot \dot{\epsilon }dt$$

and in the small-deformation regime it can be replaced by

(46) $$\underset{\eta \in {\left[0,2\pi \right)}^{N}}{\max}\;\int_{0}^{T}\dot{\epsilon }\cdot V\epsilon \;dt.$$

Denote the actuation phase differences between adjacent segments by

$$p_{n}:=\Delta \eta (n)=\eta_{n+1}-\eta_{n}\text{ for }n=1,\ldots ,N-1.$$

From observations of numerical simulations, Fang et al. (2015) report that “[…] *the optimized phase-different patterns are always reflectionally symmetric* [about the center, Ed.] *regardless of the initial symmetry requirements* […]” and of the number of segments. Thus, a solution to (45) fulfills

(47) $$p_{n}=p_{N-n}\;\forall \;n.$$

In fact these properties can be rigorously proved under the assumption that problem (45) admits a unique solution in [0, 2π)^*N*^ (see Appendix D in Supplementary Material).

Furthermore, property (47) can be proved also for (46), assuming it admits a unique solution in [0, 2π)^*N*^. To this aim, denote the unique solution to (46) by

$$\tilde{\epsilon }(t):={\left\{{\tilde{\epsilon }}_{n}(t):=a\sin\left(\frac{2\pi }{T}t+{\tilde{\eta }}_{n}\right)\right\}}_{n=1,\ldots ,N},$$

and consider the shape change $\hat{\epsilon }\left(t\right)$ associated with

$$\hat{\eta }:=-K\tilde{\eta }+2\pi$$

where

$$K:=\left[\begin{array}{c} \begin{array}{ccccc} 0 & 0 & \cdots & 0 & 1\\ 0 & 0 & \cdots & 1 & 0\\ \vdots & \vdots & \ddots & \vdots & \vdots \\ \text{0} & \text{1} & \cdots & \text{0} & \text{0}\\ \text{1} & \text{0} & \cdots & \text{0} & \text{0} \end{array} \end{array}\right]\in {\mathbb{R}}^{N\times N}\;.$$

Notice that for *n* = 1, …, *N*,

$${\hat{\epsilon }}_{n}(t):=a\sin\left(\frac{2\pi }{T}t-{\left(K\tilde{\eta }\right)}_{n}\right)={\left\{-K\tilde{\epsilon }(-t)\right\}}_{n}$$

and hence, by exploiting the fact that *V* is skew-centrosymmetric (namely, 𝕂^*T*^𝕍𝕂 = −𝕍),

$$\begin{array}{l} \int_{0}^{T}\dot{\hat{\epsilon }}\cdot V\hat{\epsilon }dt=-\int_{0}^{T}\dot{\tilde{\epsilon }}(-t)\cdot K^{T}VK\tilde{\epsilon }(-t)dt\\ \;=\int_{-T}^{0}\dot{\tilde{\epsilon }}\cdot V\tilde{\epsilon }dt=\int_{0}^{T}\dot{\tilde{\epsilon }}\cdot V\tilde{\epsilon }dt. \end{array}$$

Thus

$$\tilde{\eta }=-K\tilde{\eta }+2\pi$$

which leads to (47).

Problem (46) constrains the *L*^2^-norm of the time-derivatives, i.e., for strains having the form (43) we get

$$\int_{0}^{T}\dot{\epsilon }\cdot \dot{\epsilon }dt=\frac{2N}{T}{\left(a\pi \right)}^{2}=:c^{⋆}$$

regardless of **η**. Therefore we can extend the maximization to the *C*^2^ periodic strains whose time derivative fulfills the same constraint, i.e.,

(48) $$\begin{array}{l} \;\underset{{}_{\epsilon \in S}}{\max}\;V\left[\epsilon ,\dot{\epsilon }\right]:=\int_{0}^{T}\dot{\epsilon }\cdot V\epsilon \;dt\\ S=\{\int_{0}^{T}\epsilon \in C^{2}\left(\mathbb{R},{\mathbb{R}}^{N}\right)\;|\;\epsilon (0)=\epsilon (T)\wedge \;\int_{0}^{T}\dot{\epsilon }\cdot \dot{\epsilon }dt=c^{⋆}\}. \end{array}$$

**Figure 11:**
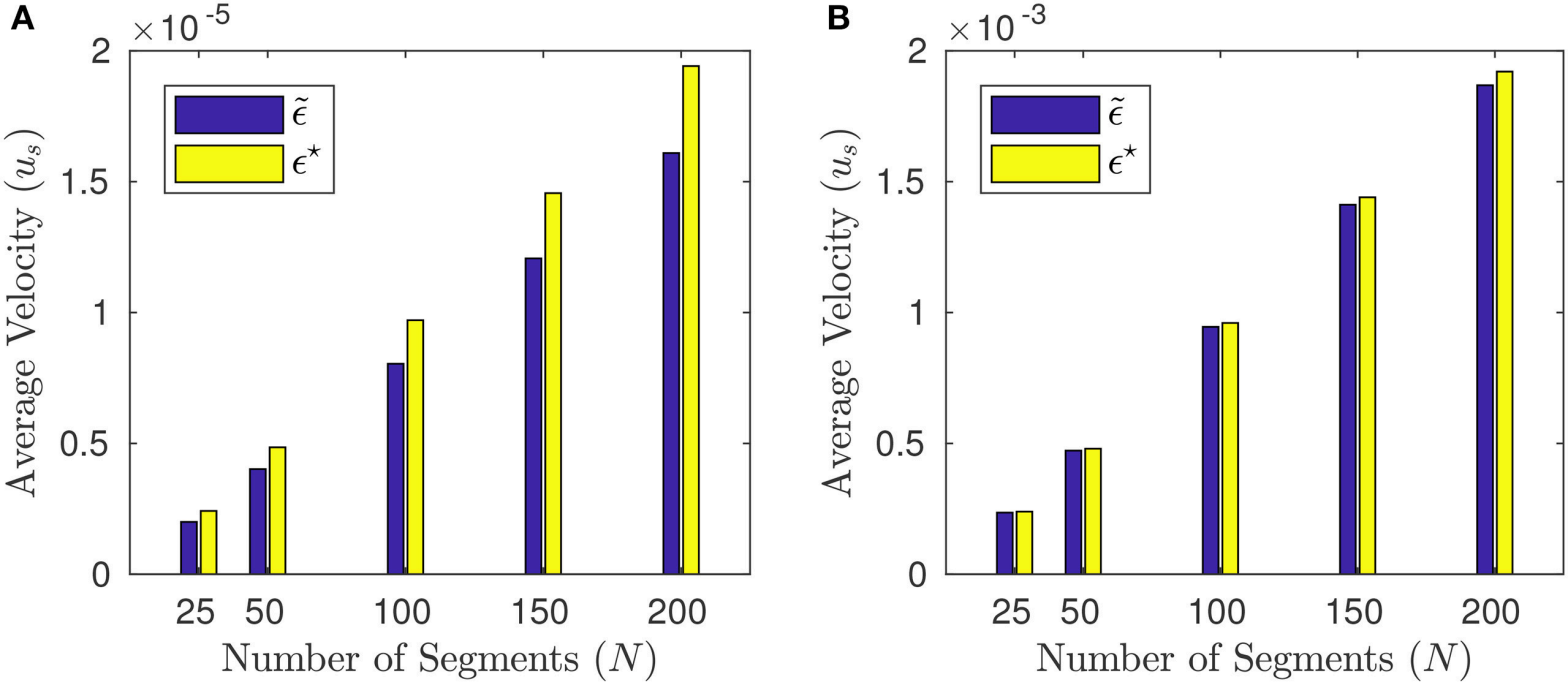
Average velocities *u*_*s*_, (44) obtained by the solution $\tilde{\epsilon }$ to (46) (blue bars) and by the solution ϵ^⋆^ to (48) (yellow bars) for different numbers of segments: *N* = 25, 50, 100, 150, 200. **(A)** is for *p* = 0 (Newtonian case) and **(B)** for *p* = 100. The other parameters are *T* = 2π, *a* = 2^−10^, *L* = 1.

Since problem (21) reduces to (48) when 𝔸 = 0 and 𝔹 = 𝕀_*N*_, a solution to (48) must be of the form

(49) $$\epsilon_{n}^{⋆}(t)=a\sqrt{N}\;||e_{n}||\;\sin\left(\frac{2\pi }{T}t+\arg\left(e_{n}\right)+\vartheta_{a}\right)$$

where **e** = (_*e*_*n*_)*n*_ is a unit eigenvector associated with the maximum-modulus eigenvalue of *V* and ϑ_*a*_ is a constant. Notice that the *reflectional symmetry* about the center still holds true. As a matter of fact, (49) leads to a slight increment in the net displacement with respect to the solution to (46), cf. Figure 11.

### 4.2. Summary and outlook

Our analysis confirms the effectiveness of mimicking peristalsis in bio-inspired robots, at least in the small-deformation regime. This bio-inspired actuation strategy has been implemented on a trial-and-error basis many times in the robotics literature and, more recently, also proposed as optimal (in some suitably defined sense, and in some suitably defined class of actuation strategies). Our main result is a mathematically rigorous proof that, in the small deformation regime, actuation by peristaltic waves is an optimal control strategy emerging naturally from the geometric symmetry of the system, namely, the invariance under shifts along the body axis. This is true exactly in the periodic case, and approximately true in the case of finite length, modulo edge-effects. Our results is of theoretical nature. Nevertheless, we believe that it has important consequences for applications. For example, it helps us not to fall into the naive temptation to expect that peristaltic waves are always an optimal actuation strategy just because they are observed in nature, but to exercise critical judgment whenever the hypotheses on the geometric symmetry that are “responsible” for the optimality result in our case (invariance under shift of a homogeneous one-dimensional system) are false.

Actuation by phase coordination, optimal actuation by identical phase difference, and the connections between this and traveling waves have been already discussed in the literature (e.g., Fang et al., 2015), but never through a mathematically rigorous analysis of the optimal control problem, of the symmetry properties of the governing equations and operators, and of the relation between these and the geometric symmetries of the system. This is exactly what we do in this paper. The added value of this analysis is that we are able to show (for the first time, to the best of our knowledge, at least in the robotics literature) that peristaltic waves are the signature of the invariance with respect to shifts (a geometric symmetry) of a homogeneous one-dimensional system.

Further work will be needed to test the effectiveness of peristaltic waves as a locomotion strategies if large deformations are allowed. In addition, future work will explore the issue of how peristalsis is actually enforced in biological systems. Of particular interest is the dichotomy between the paradigm of actuations via a Central Pattern Generator (CPG), as opposed to local sensory and feedback mechanisms. The CPG paradigm is apparent in several different organisms (Marder and Bucher, 2001; Grillner, 2006) and has been employed in robotics with some success (Ijspeert, 2008; Boxerbaum et al., 2012). However, there is a growing awareness of the role played by proprioception, especially for lower organisms such as the nematode worm *C. elegans* (Boyle et al., 2012; Wen et al., 2012) and *D. melanogaster* larvae (Pehlevan et al., 2016).

## Author contributions

AD and FA conceived research. DA executed research and performed numerical simulation. AD supervised research. FA contributed expertise on circulant matrices. All authors analyzed the data and wrote the manuscript.

### Conflict of interest statement

The authors declare that the research was conducted in the absence of any commercial or financial relationships that could be construed as a potential conflict of interest. The handling Editor declared a shared affiliation, though no other collaboration, with one of the authors, AD.

## References

[B1] Alberts B. Johnson A. Lewis J. Raff M. (2002). Molecular Biology of the Cell, 4th Edn. New York, NY: Garland Science.

[B2] Boxerbaum A. Shaw K. Chiel H. Quinn R. (2012). Continuous wave peristaltic motion in a robot. Int. J. Robot. Res. 31, 302–318. 10.1177/0278364911432486

[B3] Boyle J. H. Berri S. Cohen N. (2012). Gait modulation in c. elegans: an integrated neuromechanical model. Front. Comput. Neurosci. 6:10. 10.3389/fncom.2012.00010 22408616 PMC3296079

[B4] Collar A. (1962). On centrosymmetric and centroskew matrices. Q. J. Mechan. Appl. Math. 15, 265–281. 10.1093/qjmam/15.3.265

[B5] Daltorio K. Boxerbaum A. Horchler A. S. Shaw K. M. Chiel H. J. Quinn R. D. (2013). Efficient worm-like locomotion: slip and control of soft-bodied peristaltic robots. Bioinspir. Biomimet. 8:035003. 10.1088/1748-3182/8/3/035003 23981561

[B6] Denny M. (1980). The role of gastropod pedal mucus in locomotion. Nature 285:160 10.1038/285160a0

[B7] DeSimone A. Guarnieri F. Noselli G. Tatone A. (2013). Crawlers in viscous environments: linear vs non-linear rheology. Int. J. Non Linear Mech. 56, 142–147. 10.1016/j.ijnonlinmec.2013.02.007

[B8] DeSimone A. Tatone A. (2012). Crawling motility through the analysis of model locomotors: two case studies. Eur. Phys. J. E Soft Matt. Biol. Phys. 35, 1–8. 10.1140/epje/i2012-12085-x 22972227

[B9] Edwards C. Hendrix P. Arancon N. (2018). Biology and Ecology of Earthworms. Springer Available online at: https://www.springer.com/in/book/9780387749426

[B10] Fang H. Li S. Wang K. W. Xu J. (2015). Phase coordination and phase–velocity relationship in metameric robot locomotion. Bioinspir. Biomimet. 10:066006 10.1088/1748-3190/10/6/066006 26513696

[B11] Gardner C. (1976). The neuronal control of locomotion in the earthworm. Biol. Rev. 51, 25–52. 10.1111/j.1469-185X.1976.tb01119.x 766843

[B12] Garrey W. Moore A. (1915). Peristalsis and coordination in the earthworm. Am. J. Physiol. Legacy Cont. 39, 139–148. 10.1152/ajplegacy.1915.39.2.139

[B13] Ge J. Calderón A. Pérez-Arancibia N. (2017). An earthworm-inspired soft crawling robot controlled by friction. arXiv [preprint] arXiv:1707.04084.

[B14] Gray J. Lissmann H. (1938). Studies in animal locomotion: Vii. locomotory reflexes in the earthworm. J. Exp. Biol. 15, 506–517.

[B15] Grillner S. (2006). Biological pattern generation: the cellular and computational logic of networks in motion. Neuron 52, 751–766. 10.1016/j.neuron.2006.11.008 17145498

[B16] Ijspeert A. (2008). Central pattern generators for locomotion control in animals and robots: a review. Neural Netw. 21, 642–653. 10.1016/j.neunet.2008.03.014 18555958

[B17] Marder E. Bucher D. (2001). Central pattern generators and the control of rhythmic movements. Curr. Biol. 11, R986–R996. 10.1016/S0960-9822(01)00581-4 11728329

[B18] McInerney A. (2013). First Steps in Differential Geometry: Riemannian, Contact, Symplectic. Undergraduate Texts in Mathematics. New York, NY: Springer.

[B19] Menciassi A. Accoto D. Gorini S. Dario P. (2006). Development of a biomimetic miniature robotic crawler. Auton. Robots 21, 155–163. 10.1007/s10514-006-7846-9

[B20] Nemitz M. P. Mihaylov P. Barraclough T. W. Ross D. Stokes A. A. (2016). Using voice coils to actuate modular soft robots: wormbot, an example. Soft Robot. 3, 198–204. 10.1089/soro.2016.0009 28078195 PMC5180079

[B21] Pehlevan C. Paoletti P. Mahadevan L. (2016). Integrative neuromechanics of crawling in *D. melanogaster* larvae. Elife 5:23 10.7554/eLife.11031 PMC4959844 42601672

[B22] Quillin K. (1999). Kinematic scaling of locomotion by hydrostatic animals: ontogeny of peristaltic crawling by the earthworm lumbricus terrestris. J. Exp. Biol. 202, 661–674. 10021320 10.1242/jeb.202.6.661

[B23] Umedachi T. Kano T. Ishiguro A. Trimmer B. (2016). Gait control in a soft robot by sensing interactions with the environment using self-deformation. R. Soc. Open Sci. 3:160766. 10.1098/rsos.160766 28083114 PMC5210696

[B24] Van Brunt B. (2004). The Calculus of Variations. New York, NY: Springer.

[B25] Wang K. Yan G. Ma G. Ye D. (2009). An earthworm-like robotic endoscope system for human intestine: design, analysis, and experiment. Anna. Biomed. Eng. 37, 210–221. 10.1007/s10439-008-9597-6 19003537

[B26] Wen Q. Po M. D. Hulme E. Chen S. Liu X. Kwok S. W.. (2012). Proprioceptive coupling within motor neurons drives c. elegans forward locomotion. Neuron 76, 750–761. 10.1016/j.neuron.2012.08.039 23177960 PMC3508473

[B27] Wiezel O. Giraldi L. DeSimone A. Or Y. Alouges F. (2018). Energy-optimal small-amplitude strokes for multi-link microswimmers: Purcell's loops and taylor's waves reconciled. arXiv [preprint] arXiv:1801.04687.

