# Supplementary material for: Peristaltic Waves as Optimal Gaits in Metameric Bio-Inspired Robots
Source: Front Robot AI. 2018 Sep 5;5:99. doi: 10.3389/frobt.2018.00099 (PMC7806059; doi:10.3389/frobt.2018.00099)
Supplement: Supplementary file 1 [file Data_Sheet_1.pdf]

APPENDIX A
THE DISPLACEMENT IN THE SMALL-DEFORMATION REGIME

Since

$$\dot{x}_0 = \sum_{n=1}^{N} v_n(\boldsymbol{\epsilon})\dot{\epsilon}_n \quad \text{where} \quad v_n(\boldsymbol{\epsilon}) := -\frac{L}{2}\frac{(1+\epsilon_n)^{1-p} + 2\sum\limits_{j=n+1}^{N}(1+\epsilon_j)^{1-p}}{\sum\limits_{j=1}^{N}(1+\epsilon_j)^{1-p}}\,, \tag{1}$$

we have

$$\{v_{\boldsymbol{\epsilon}}(\boldsymbol{\epsilon})\}_{ij} := \frac{\partial v_i}{\partial \epsilon_j} = \begin{cases} -\frac{L}{2}(1-p)\frac{2\sum_{n=1}^{N}(1+\epsilon_n)^{1-p} - \left((1+\epsilon_i)^{1-p} + 2\sum_{n=i+1}^{N}(1+\epsilon_n)^{1-p}\right)}{(1+\epsilon_j)^p\left(\sum_{n=1}^{N}(1+\epsilon_n)^{1-p}\right)^2} & \text{if } i<j \\ -\frac{L}{2}(1-p)\frac{\sum_{n=1}^{N}(1+\epsilon_n)^{1-p} - \left((1+\epsilon_i)^{1-p} + 2\sum_{n=i+1}^{N}(1+\epsilon_n)^{1-p}\right)}{(1+\epsilon_j)^p\left(\sum_{n=1}^{N}(1+\epsilon_n)^{1-p}\right)^2} & \text{if } i=j \\ \frac{L}{2}(1-p)\frac{(1+\epsilon_i)^{1-p} + 2\sum_{n=i+1}^{N}(1+\epsilon_n)^{1-p}}{(1+\epsilon_j)^p\left(\sum_{n=1}^{N}(1+\epsilon_n)^{1-p}\right)^2} & \text{if } i>j \end{cases}$$

whence

$$\{v_{\boldsymbol{\epsilon}}(\mathbf{0})\}_{ij} = \begin{cases} L(p-1)\frac{2i-1}{2N^2} & \text{if } i<j \\ L(p-1)\frac{2i-N-1}{2N^2} & \text{if } i=j \\ L(p-1)\frac{2i-2N-1}{2N^2} & \text{if } i>j \end{cases}.$$

Since $\mathbb{V}$ is the skew-symmetric part of $v_{\boldsymbol{\epsilon}}(\mathbf{0})$, we have

$$\{\mathbb{V}\}_{ij} = \begin{cases} L(p-1)\frac{i-j+N}{2N^2} & \text{if } i<j \\ 0 & \text{if } i=j \\ -L(p-1)\frac{j-i+N}{2N^2} & \text{if } i>j \end{cases} \tag{2}$$

which is a Toeplitz matrix, indeed

$$\{\mathbb{V}\}_{(i+1)(j+1)} = \{\mathbb{V}\}_{ij} \quad \forall i,j \in \{1,\dots,N-1\}$$

i.e., each descending diagonal from left to right is constant. Therefore, the matrix $\mathbb{V}$ turns out to be "skew-centrosymmetric", i.e., skew-symmetric about its center or, equivalently,

$$\{\mathbb{V}\}_{ij} = -\{\mathbb{V}\}_{(N+1-i)(N+1-j)} \qquad \forall i,j = 1,\dots,N\,.$$

APPENDIX B
EULER-LAGRANGE EQUATIONS

The Euler-Lagrange equations associated with the isoperimetric optimization problem (20) in the main text, i.e.,

$$\begin{gathered}\max_{\boldsymbol{\epsilon}\in\mathcal{S}} \quad V[\boldsymbol{\epsilon},\dot{\boldsymbol{\epsilon}}] := \int_0^T \dot{\boldsymbol{\epsilon}}\cdot\mathbb{V}\boldsymbol{\epsilon}\,dt \\ \mathcal{S} = \left\{\boldsymbol{\epsilon}\in C^2\left(\mathbb{R},\mathbb{R}^N\right) \;\middle|\; \boldsymbol{\epsilon}(0)=\boldsymbol{\epsilon}(T) \;\;\wedge\;\; \int_0^T \left(\mathbb{A}\boldsymbol{\epsilon}\cdot\boldsymbol{\epsilon}+\mathbb{B}\dot{\boldsymbol{\epsilon}}\cdot\dot{\boldsymbol{\epsilon}}\right)\,dt \equiv c\right\},\end{gathered} \tag{3}$$

led to the system of second order linear ODEs

$$\mathbb{V}\dot{\boldsymbol{\epsilon}} = \lambda\left(\mathbb{B}\ddot{\boldsymbol{\epsilon}} - \mathbb{A}\boldsymbol{\epsilon}\right) \tag{4}$$

where $\mathbb{V}$ is Toeplitz and skew-symmetric while $\mathbb{A}$ and $\mathbb{B}$ are supposed to be symmetric and positive definite.

In the following subsections we address problem (3) for two particular cases.

B1. Solutions for $\mathbb{A}\equiv 0$

For $\mathbb{A}\equiv 0$, equation (4) becomes

$$\mathbb{V}\dot{\boldsymbol{\epsilon}} = \lambda\,\mathbb{B}\ddot{\boldsymbol{\epsilon}}\,. \tag{5}$$

The strategy is to decompose (5) along the eigen-elements of

$$\mathbb{M} := \mathbb{B}^{-\frac{1}{2}}\;\mathbb{V}\;\mathbb{B}^{-\frac{1}{2}}$$

which is supposed to have N distinct eigenvalues for simplicity, in fact it would be sufficient to assume that the eigenspaces associated with the maximum-modulus eigenvalues have dimension 1. In particular, $\mathbb{M}$ is a skew-symmetric matrix and, as such, its eigenvalues are purely imaginary and, a part from 0, they go by pairs since to every purely imaginary eigenvalue there corresponds its conjugate (with the same multiplicity). This implies that 0 is an eigenvalue of $\mathbb{M}$ if and only if N is odd.
For the sake of clarity, let us assume that N is odd (if N is even the same argument can be applied by neglecting the eigenvector associated with 0). Thus, consider

$$\mathbf{v}_j^{\pm} \qquad \text{for } \; j=1,\ldots,\lfloor\tfrac{N}{2}\rfloor =: N^\star$$

(complex and orthonormal) eigenvectors associated with the purely imaginary eigenvalue

$$\pm i\mu_j \qquad \text{with } \; \mu_j > 0$$

and $\mathbf{v}_0$, eigenvector associated with $\mu_0 = 0$, so that

$$\begin{gathered}\mathbb{M}\mathbf{v}_0 = \mathbf{0} \\ \mathbb{M}\mathbf{v}_j^{\pm} = \pm i\mu_j\mathbf{v}_j^{\pm} \qquad \text{for } \; j=1,\ldots,N^\star\,.\end{gathered}$$

Therefore

$$\mathbb{B}^{\frac{1}{2}}\boldsymbol{\epsilon}(t) = \sum_{j=1}^{N^\star}\left(\psi_j^+(t)\mathbf{v}_j^+ + \psi_j^-(t)\mathbf{v}_j^-\right) + \psi_0(t)\mathbf{v}_0$$

and from (5) we get

$$\begin{cases} \lambda\ddot{\psi}_j^\pm = \pm i\mu_j\dot{\psi}_j^\pm & \text{for } j=1,\dots,N^\star \\ \lambda\ddot{\psi}_0 = 0 \end{cases}$$

whence

$$\begin{cases} \psi_j^\pm(t) = \frac{\lambda\alpha_j^\pm}{\pm i\mu_j}e^{\pm i\frac{\mu_j}{\lambda}t} + \gamma_j^\pm & \text{for } j=1,\dots,N^\star \\ \psi_0(t) = \alpha_0 t + \gamma_0 \end{cases}$$

where $\alpha_j^\pm$, $\gamma_j^\pm$, α_0 and γ_0 are complex constants; in particular, the constants $\gamma_j^\pm$ and γ_0 determine the initial condition $\boldsymbol{\epsilon}(0)$ and hence, for simplicity, we can assume that $\gamma_0 = 0$ and $\gamma_j^\pm = 0$ for $j = 1,\dots,N^\star$. Therefore, up to a constant, a solution to (5) can be written as

$$\boldsymbol{\epsilon}(t) = \sum_{j=1}^{N^\star}\left(\frac{\lambda\alpha_j^+}{i\mu_j}e^{i\frac{\mu_j}{\lambda}t}\mathbb{B}^{-\frac{1}{2}}\boldsymbol{v}_j^+ - \frac{\lambda\alpha_j^-}{i\mu_j}e^{-i\frac{\mu_j}{\lambda}t}\mathbb{B}^{-\frac{1}{2}}\boldsymbol{v}_j^-\right) + \alpha_0 t\mathbb{B}^{-\frac{1}{2}}\boldsymbol{v}_0 \qquad \text{for } t\in[0,T]\ .$$

Moreover, since a solution to (3) must be periodic, i.e., $\boldsymbol{\epsilon}(0) = \boldsymbol{\epsilon}(T)$, it turns out that

$$\begin{cases} \alpha_0 = 0 \\ \lambda = \frac{\mu_j T}{2\pi k_j} \quad \text{where } k_j\in\mathbb{N} \quad \text{for } j=1,\dots,N^\star \end{cases}.$$

On the other hand,

$$V[\boldsymbol{\epsilon},\dot{\boldsymbol{\epsilon}}] = -\int_0^T \mathbb{V}\dot{\boldsymbol{\epsilon}}\cdot\boldsymbol{\epsilon}\,dt = -\int_0^T \lambda\mathbb{B}\ddot{\boldsymbol{\epsilon}}\cdot\boldsymbol{\epsilon}\,dt = \lambda\int_0^T \mathbb{B}\dot{\boldsymbol{\epsilon}}\cdot\dot{\boldsymbol{\epsilon}}\,dt = \lambda E\left[\boldsymbol{\epsilon},\dot{\boldsymbol{\epsilon}}\right]$$

where $E\left[\boldsymbol{\epsilon},\dot{\boldsymbol{\epsilon}}\right] = c$ is constrained by the optimization problem. Then maximizing the approximated displacement leads to take λ as big as possible i.e.,

$$\lambda = \frac{\mu_M T}{2\pi} \quad \text{where } \mu_M := \max_{j=1,\dots,N^\star}\mu_j$$

and, in order to preserve the periodicity,

$$\alpha_j^\pm = 0 \qquad \text{for } j\neq M$$

yielding to

$$\boldsymbol{\epsilon}(t) = \frac{\alpha_M^+ e^{\frac{2i\pi t}{T}}}{\frac{2i\pi}{T}}\mathbb{B}^{-\frac{1}{2}}\mathbf{v}_M^+ - \frac{\alpha_M^- e^{-\frac{2i\pi t}{T}}}{\frac{2i\pi}{T}}\mathbb{B}^{-\frac{1}{2}}\mathbf{v}_M^-$$

where $\mathbf{v}_M^-$ is the conjugate of $\mathbf{v}_M^+$ and, since $\boldsymbol{\epsilon}(t)$ must be in $\mathbb{R}^N$, α_M^+ is the conjugate of α_M^- and needs to fulfill

$$||\alpha_M^+|| = \sqrt{\frac{c}{2T}} \tag{6}$$

indeed

$$E[\boldsymbol{\epsilon},\dot{\boldsymbol{\epsilon}}] = T\sum_{j=1}^{N^\star}\left(||\alpha_j^+||^2 + ||\alpha_j^-||^2\right) = 2T||\alpha_M^+||^2\,.$$

Therefore we can finally conclude that a solution to (5) has the form

$$\boldsymbol{\epsilon}(t) = \frac{\alpha e^{\frac{2i\pi t}{T}}}{\frac{2i\pi}{T}}\mathbb{B}^{-\frac{1}{2}}\mathbf{v} - \frac{\bar{\alpha} e^{-\frac{2i\pi t}{T}}}{\frac{2i\pi}{T}}\mathbb{B}^{-\frac{1}{2}}\bar{\mathbf{v}}$$

where the bar denotes complex conjugation. Finally, this expression can be rewritten as

$$\boldsymbol{\epsilon}(t) = -\frac{T}{\pi}\Re\left(\alpha i e^{\frac{2i\pi t}{T}}\mathbf{e}\right) \qquad \text{where} \quad \mathbf{e} := \mathbb{B}^{-\frac{1}{2}}\mathbf{v} \quad \text{and} \quad ||\alpha|| = \sqrt{\frac{c}{2T}}. \tag{7}$$

Notice that for maximizing the displacement in the opposite direction we should consider

$$\lambda = -\frac{\mu_M T}{2\pi} \quad \text{where } \mu_M := \max_{j=1,\dots,N^\star}\mu_M$$

so that (7) becomes

$$\boldsymbol{\epsilon}(t) = \frac{T}{\pi}\Re\left(\alpha i e^{-\frac{2i\pi t}{T}}\mathbf{e}\right)\,.$$

B2. Solutions for $\mathbb{B} \equiv 0$

For $\mathbb{B} \equiv 0$, equation (4) becomes

$$\mathbb{V}\dot{\boldsymbol{\epsilon}} = -\lambda\,\mathbb{A}\boldsymbol{\epsilon}\,. \tag{8}$$

The strategy is, as before, to decompose (8) along the eigen-elements of

$$\mathbb{M} := \mathbb{A}^{-\frac{1}{2}}\;\mathbb{V}\;\mathbb{A}^{-\frac{1}{2}}$$

which is supposed to have N distinct eigenvalues for simplicity, in fact it would be sufficient to assume that the eigenspaces associated with the maximum-modulus eigenvalues have dimension 1. In particular, $\mathbb{M}$ is a skew-symmetric matrix and, as such, its eigenvalues are purely imaginary and, a part from 0, they go by pairs since to every purely imaginary eigenvalue there corresponds its conjugate (with the same multiplicity). This implies that 0 is an eigenvalue of $\mathbb{M}$ if and only if N is odd.
For the sake of clarity, let us assume that N is odd (if N is even the same argument can be applied by neglecting the eigenvector associated with 0). Thus, consider

$$\mathbf{v}_j^\pm \qquad \text{for } j = 1,\dots,\lfloor \tfrac{N}{2}\rfloor =: N^\star$$

(complex and orthonormal) eigenvectors associated with the purely imaginary eigenvalue

$$\pm i\mu_j \qquad \text{with } \mu_j > 0$$

and $\mathbf{v}_0$, eigenvector associated with $\mu_0 := 0$, so that

$$\begin{aligned}\mathbb{M}\mathbf{v}_0 &= \mathbf{0}\\ \mathbb{M}\mathbf{v}_j^{\pm} &= \pm i\mu_j \mathbf{v}_j^{\pm} \qquad \text{for } j = 1, \dots, N^\star .\end{aligned}$$

Therefore

$$\mathbb{A}^{\frac{1}{2}}\boldsymbol{\epsilon}(t) = \sum_{j=1}^{N^\star} \left(\psi_j^+(t)\mathbf{v}_j^+ + \psi_j^-(t)\mathbf{v}_j^-\right) + \psi_0(t)\mathbf{v}_0$$

and from (8) we get

$$\begin{cases}\pm i\mu_j \dot{\psi}_j^{\pm} = -\lambda \psi_j^{\pm} & \text{for } j = 1, \dots, N^\star\\ \lambda\psi_0 = 0\end{cases}$$

whence

$$\begin{cases}\psi_j^{\pm}(t) = \alpha_j^{\pm} e^{\frac{\pm i\lambda}{\mu_j}t} & \text{for } j = 1, \dots, N^\star\\ \psi_0(t) \equiv 0\end{cases}$$

where $\alpha_j^{\pm}$ are complex constants. Therefore a solution to (8) can be written as

$$\boldsymbol{\epsilon}(t) = \sum_{j=1}^{N^\star} \left(\alpha_j^+ e^{\frac{i\lambda}{\mu_j}t} \mathbb{A}^{-\frac{1}{2}} \boldsymbol{v}_j^+ - \alpha_j^- e^{-\frac{i\lambda}{\mu_j}t} \mathbb{A}^{-\frac{1}{2}} \boldsymbol{v}_j^-\right) \qquad \text{for } t \in [0, T] .$$

Moreover, since a solution to (3) must be periodic, i.e., $\boldsymbol{\epsilon}(0) = \boldsymbol{\epsilon}(T)$, it turns out that

$$\lambda = \frac{2\pi k_j \mu_j}{T} \quad \text{where } k_j \in \mathbb{N} \quad \text{for } j = 1, \dots, N^\star .$$

On the other hand,

$$V[\boldsymbol{\epsilon}, \dot{\boldsymbol{\epsilon}}] = -\int_0^T \mathbb{V}\dot{\boldsymbol{\epsilon}} \cdot \boldsymbol{\epsilon}\, dt = \lambda \int_0^T \mathbb{A}\boldsymbol{\epsilon} \cdot \boldsymbol{\epsilon}\, dt = \lambda E\left[\boldsymbol{\epsilon}, \dot{\boldsymbol{\epsilon}}\right]$$

where $E\left[\boldsymbol{\epsilon}, \dot{\boldsymbol{\epsilon}}\right] = c$ is constrained by the optimization problem. Then maximizing the approximated displacement leads to take λ as big as possible but, since in principle k_j could tend to infinity, in order to have a meaningful problem, we restrict our attention to shape changes with unitary time frequency (one wave per period), that is $k_j = 1$ and hence

$$\lambda = \frac{2\pi\mu_M}{T} \quad \text{where } \mu_M := \max_{j=1,\dots,N^\star} \mu_M .$$

In order to preserve the periodicity,

$$\alpha_j^{\pm} = 0 \qquad \text{for } j \neq M$$

yielding to

$$\boldsymbol{\epsilon}(t) = \alpha_M^+ e^{\frac{2i\pi t}{T}} \mathbb{B}^{-\frac{1}{2}} \mathbf{v}_M^+ - \alpha_M^- e^{-\frac{2i\pi t}{T}} \mathbb{B}^{-\frac{1}{2}} \mathbf{v}_M^-$$

where $\mathbf{v}_M^-$ is the conjugate of $\mathbf{v}_M^+$ and, since $\boldsymbol{\epsilon}(t)$ must be in $\mathbb{R}^N$, α_M^+ is the conjugate of α_M^- and needs to fulfill

$$||\alpha_M^+|| = \sqrt{\frac{c}{2T}} \tag{9}$$

indeed

$$E\left[\boldsymbol{\epsilon},\dot{\boldsymbol{\epsilon}}\right] = T\sum_{j=1}^{N^\star}\left(||\alpha_j^+||^2 + ||\alpha_j^-||^2\right) = 2T||\alpha_M^+||^2 \,.$$

We conclude that a solution to (8) has the form

$$\boldsymbol{\epsilon}(t) = \alpha e^{\frac{2i\pi t}{T}}\mathbb{B}^{-\frac{1}{2}}\mathbf{v} - \bar{\alpha}e^{-\frac{2i\pi t}{T}}\mathbb{B}^{-\frac{1}{2}}\bar{\mathbf{v}}$$

where the bar denotes complex conjugation. This expression can be rewritten as

$$\boldsymbol{\epsilon}(t) = 2\Re\left(\alpha e^{\frac{2i\pi t}{T}}\mathbf{e}\right) \qquad \text{where} \quad \mathbf{e} := \mathbb{B}^{-\frac{1}{2}}\mathbf{v} \quad \text{and} \quad ||\alpha|| = \sqrt{\frac{c}{2T}}. \tag{10}$$

Notice that for maximizing the displacement in the opposite direction we should consider

$$\lambda = -\frac{2\pi\mu_M}{T} \quad \text{where } \mu_M := \max_{j=1,\dots,N^\star}\mu_M$$

so that the solution has the form

$$\boldsymbol{\epsilon}(t) = 2\Re\left(\alpha e^{-\frac{2i\pi t}{T}}\mathbf{e}\right)\,.$$

B3. Symmetry properties

Consider

$$E[\boldsymbol{\epsilon},\dot{\boldsymbol{\epsilon}}] := \int_0^T\left(\mathbb{A}\boldsymbol{\epsilon}\cdot\boldsymbol{\epsilon} + \mathbb{B}\dot{\boldsymbol{\epsilon}}\cdot\dot{\boldsymbol{\epsilon}}\right)\,dt$$

where $\mathbb{A}\equiv 0$ and B is centrosymmetric (resp. A is centrosymmetric and $\mathbb{B}\equiv 0$).
As a well-known result about centrosymmetric and skew-centrosymmetric matrices (e.g., Collar, 1962),

$$\mathbb{K}^T\mathbb{B}\mathbb{K} = \mathbb{B} \qquad (\text{resp. } \mathbb{K}^T\mathbb{A}\mathbb{K} = \mathbb{A})$$

and

$$\mathbb{K}^T\mathbb{V}\mathbb{K} = -\mathbb{V}\,,$$

where

$$\mathbb{K} := \begin{bmatrix} 0 & 0 & \cdots & 0 & 1 \\ 0 & 0 & \cdots & 1 & 0 \\ \vdots & \vdots & \iddots & \vdots & \vdots \\ 0 & 1 & \cdots & 0 & 0 \\ 1 & 0 & \cdots & 0 & 0 \end{bmatrix} \in \mathbb{R}^{N\times N}\,.$$

From the previous subsections, a solution to (3) has the form

$$\boldsymbol{\epsilon}^\star(t) = -\frac{T}{\pi}\Re\left(\alpha i e^{\frac{2i\pi t}{T}}\mathbf{e}\right) \qquad \left(\text{resp. } \boldsymbol{\epsilon}^\star(t) = 2\Re\left(\alpha e^{\frac{2i\pi t}{T}}\mathbf{e}\right)\right) \qquad \text{where} \quad ||\alpha|| = \sqrt{\frac{c}{2T}}\,.$$

Notice that

$$\boldsymbol{\eta}^\star(t) := \mathbb{K}\boldsymbol{\epsilon}^\star(t) = -\frac{T}{\pi}\Re\left(\alpha i e^{\frac{2i\pi t}{T}}\mathbb{K}\mathbf{e}\right) \qquad \left(\text{resp. } \boldsymbol{\eta}^\star(t) := \mathbb{K}\boldsymbol{\epsilon}^\star(t) = 2\Re\left(\alpha e^{\frac{2i\pi t}{T}}\mathbb{K}\mathbf{e}\right)\right)$$

is a solution to

$$\min_{\boldsymbol{\epsilon}\in\mathcal{S}} \quad -V[\boldsymbol{\epsilon},\dot{\boldsymbol{\epsilon}}] := -\int_0^T \dot{\boldsymbol{\epsilon}}\cdot\mathbb{V}\boldsymbol{\epsilon}\,dt\,. \tag{11}$$

Indeed

$$E[\boldsymbol{\eta}^\star,\boldsymbol{\eta}^\star] = E[\boldsymbol{\epsilon}^\star,\dot{\boldsymbol{\epsilon}}^\star] = c$$

and

$$V[\boldsymbol{\eta}^\star,\boldsymbol{\eta}^\star] = \int_0^T \mathbb{K}\dot{\boldsymbol{\epsilon}}^\star\cdot\mathbb{V}\mathbb{K}\boldsymbol{\epsilon}^\star\,dt = -\int_0^T \dot{\boldsymbol{\epsilon}}^\star\cdot\mathbb{V}\boldsymbol{\epsilon}^\star\,dt = -V[\boldsymbol{\epsilon}^\star,\dot{\boldsymbol{\epsilon}}^\star] = -\max_{\boldsymbol{\epsilon}\in\mathcal{S}} V[\boldsymbol{\epsilon},\dot{\boldsymbol{\epsilon}}] = \min_{\boldsymbol{\epsilon}\in\mathcal{S}}\left(-V[\boldsymbol{\epsilon},\dot{\boldsymbol{\epsilon}}]\right)\,.$$

Since $\boldsymbol{\eta}^\star(t)$ is a solution to (11), it must be of the form

$$\boldsymbol{\eta}^\star(t) = \frac{T}{\pi}\Re\left(\beta i e^{-\frac{2i\pi t}{T}}\mathbf{e}\right) \qquad \left(\text{resp. } \boldsymbol{\eta}^\star(t) = 2\Re\left(\beta e^{-\frac{2i\pi t}{T}}\mathbf{e}\right)\right) \qquad \text{where} \quad ||\beta|| = \sqrt{\frac{c}{2T}}\,.$$

Therefore

$$-\Re\left(\alpha i e^{\frac{2i\pi t}{T}}\mathbb{K}\mathbf{e}\right) = \Re\left(\beta i e^{-\frac{2i\pi t}{T}}\mathbf{e}\right) \qquad \left(\text{resp. } \Re\left(\alpha e^{\frac{2i\pi t}{T}}\mathbb{K}\mathbf{e}\right) = \Re\left(\beta e^{-\frac{2i\pi t}{T}}\mathbf{e}\right)\right) \qquad \forall t\in[0,T]\,,$$

namely, for $n = 1,\dots,N$, the real parts of $i\beta e_n$ and $-i\alpha(\mathbb{K}\mathbf{e})_n$ (resp. βe_n and $\alpha(\mathbb{K}\mathbf{e})_n$) coincide for any simultaneous opposite rotation (i.e., multiplication by $e^{-\frac{2i\pi t}{T}}$ and $e^{\frac{2i\pi t}{T}}$ $\forall t\in[0,T]$), whence

$$\mathbb{K}\mathbf{e} = e^{i\vartheta}\bar{\mathbf{e}}$$

for some suitable $\vartheta\in[0,2\pi)$. In particular,

$$e_{N+1-n} = e^{i\vartheta}\bar{e}_n \quad \forall\, n = 1,\dots,N$$

and hence

- moduli are symmetric about the center, i.e.,

$$||e_{N+1-n}|| = ||e_n|| \quad \forall\, n = 1,\dots,N\,;$$

- phase differences between adjacent segments are symmetric about the center, i.e.,

$$e_{n+1}e_{N+1-n} = e_{N-n}e_n \quad \forall\, n = 1,\dots,N\,.$$

APPENDIX C
DISSIPATION

C1. The first term of the dissipation rate: the power

By definition,

$$d_1(t,\boldsymbol{\epsilon},\dot{\boldsymbol{\epsilon}}) := \int_0^{NL} -\tfrac{1}{\mu} f\left(x_0(t)+s(X,t),t\right)\ s'(X,t)\ \dot{\chi}(X,t)\,dX$$

where, for $X \in [X_{n-1} := (n-1)L, X_n := nL]$ (i.e., n-th segment),

$$\dot{\chi}(X,t) = \dot{x_0}(t) + \dot{s}(X,t) = \dot{x_0}(t) + [X-(n-1)L]\,\dot{\epsilon}_n(t) + L\sum_{i=1}^{n-1}\dot{\epsilon}_i(t)\,,$$

$$f\left(x_0(t)+s(X,t),t\right) = (1+\epsilon(X,t))^{-p}\dot{\chi}(X,t)$$

$$s'(X,t) = 1+\epsilon(X,t)\ .$$

Therefore,

$$\begin{aligned} d_1(t,\boldsymbol{\epsilon},\dot{\boldsymbol{\epsilon}}) &= \int_0^{NL} (1+\epsilon(X,t))^{1-p}\,\dot{\chi}^2(X,t)\,dX \\ &= \int_0^{NL} (1+\epsilon(X,t))^{1-p}\left[\dot{x_0}(t) + (X-(n-1)L)\,\dot{\epsilon}_n(t) + L\sum_{i=1}^{n-1}\dot{\epsilon}_i(t)\right]^2 dX = \sum_{n=1}^{N} D_n \end{aligned}$$

where, for all $n = 1,\ldots,N$,

$$\begin{aligned} D_n &:= \int_{(n-1)L}^{nL} (1+\epsilon_n)^{1-p}\left[\dot{x_0} + L\sum_{i=1}^{n-1}\dot{\epsilon}_i + (X-(n-1)L)\,\dot{\epsilon}_n\right]^2 dX \\ &= (1+\epsilon_n)^{1-p}\int_{(n-1)L}^{nL}\Bigg\{\left[\dot{x_0}+L\sum_{i=1}^{n-1}\dot{\epsilon}_i\right]^2 + 2\left[\dot{x_0}+L\sum_{i=1}^{n-1}\dot{\epsilon}_i\right](X-(n-1)L)\,\dot{\epsilon}_n \\ &\quad + \dot{\epsilon_n}^2\,(X-(n-1)L)^2\Bigg\}\,dX \\ &= (1+\epsilon_n)^{1-p}\Bigg\{\left[\dot{x_0}+L\sum_{i=1}^{n-1}\dot{\epsilon}_i\right]^2 L + \left[\dot{x_0}+L\sum_{i=1}^{n-1}\dot{\epsilon}_i\right]\dot{\epsilon}_n\,(X-(n-1)L)^2\Bigg|_{(n-1)L}^{nL} \\ &\quad + \dot{\epsilon_n}^2\,\frac{(X-(n-1)L)^3}{3}\Bigg|_{(n-1)L}^{nL}\Bigg\} \\ &= \frac{L}{3}(1+\epsilon_n)^{1-p}\left\{3\left[\dot{x_0}+L\sum_{i=1}^{n-1}\dot{\epsilon}_i\right]^2 + 3L\left[\dot{x_0}+L\sum_{i=1}^{n-1}\dot{\epsilon}_i\right]\dot{\epsilon}_n + \dot{\epsilon_n}^2 L^2\right\}\ . \end{aligned}$$

In view of (1),

$$\dot{x_0} + L\sum_{i=1}^{n-1}\dot{\epsilon}_i = \sum_{n=1}^{N} v_n\dot{\epsilon}_n + L\sum_{i=1}^{n-1}\dot{\epsilon}_i = \sum_{i=1}^{n-1}(L+v_i)\dot{\epsilon}_i + \sum_{i=n}^{N} v_i\dot{\epsilon}_i \qquad \forall n = 1,\dots,N$$

whence

$$\begin{aligned}
D_n &= \frac{L}{3}\left(1+\epsilon_n\right)^{1-p}\left\{3\left[\sum_{j=1}^{n-1}(L+v_j)\dot{\epsilon}_j + \sum_{i=n}^{N} v_j\dot{\epsilon}_j\right]^2 + 3L\left[\sum_{j=1}^{n-1}(L+v_j)\dot{\epsilon}_j + \sum_{j=n}^{N} v_j\dot{\epsilon}_j\right]\dot{\epsilon}_n + \dot{\epsilon_n}^2L^2\right\} \\
&= \frac{L}{3}\left(1+\epsilon_n\right)^{1-p}\left\{3\left[\sum_{j=1}^{n-1}(L+v_j)^2\dot{\epsilon}_j^2 + 2\sum_{\substack{i,j=1\\ i<j}}^{n-1}(L+v_j)(L+v_i)\dot{\epsilon}_i\dot{\epsilon}_j + \sum_{j=n}^{N} v_j^2\dot{\epsilon}_j^2 + 2\sum_{\substack{i,j=n\\ i<j}}^{N} v_iv_j\dot{\epsilon}_i\dot{\epsilon}_j\right.\right. \\
&\qquad \left.\left. + 2\sum_{i=1}^{n-1}\sum_{j=n}^{N}(L+v_i)v_j\dot{\epsilon}_i\dot{\epsilon}_j\right] + 3L\left[\sum_{j=1}^{n-1}(L+v_j)\dot{\epsilon}_j + \sum_{j=n}^{N} v_j\dot{\epsilon}_j\right]\dot{\epsilon}_n + \dot{\epsilon_n}^2L^2\right\} \\
&= \frac{L}{3}\left(1+\epsilon_n\right)^{1-p}\left\{L^2\dot{\epsilon}_n^2 + 3\sum_{j=1}^{n-1}(L+v_j)^2\dot{\epsilon_j}^2 + 3\sum_{j=n}^{N} v_j^2\dot{\epsilon}_j^2 + 3L\sum_{j=1}^{n-1}(L+v_j)\dot{\epsilon}_j\dot{\epsilon}_n + 3L\sum_{j=n}^{N} v_j\dot{\epsilon}_j\dot{\epsilon}_n\right. \\
&\qquad \left. +2\sum_{\substack{i,j=1\\ i<j}}^{n-1} 3(L+v_j)(L+v_i)\dot{\epsilon}_i\dot{\epsilon}_j + 2\sum_{\substack{i,j=n\\ i<j}}^{N} 3v_iv_j\dot{\epsilon}_i\dot{\epsilon}_j + 2\sum_{j=n}^{N}\sum_{i=1}^{n-1} 3(L+v_i)v_j\dot{\epsilon}_i\dot{\epsilon}_j\right\} \\
&= \frac{L}{3}\left(1+\epsilon_n\right)^{1-p}\left\{\sum_{j=1}^{N} a_j^{(n)}(\boldsymbol{\epsilon})\dot{\epsilon}_j^2 + \dot{\epsilon}_j^2 + 2\sum_{\substack{j=1\\ j\neq n}}^{N} b_j^{(n)}(\boldsymbol{\epsilon})\dot{\epsilon}_j\dot{\epsilon}_n + 2\sum_{\substack{i,j=1\\ i<j}}^{n-1} c_{ij}(\boldsymbol{\epsilon})\dot{\epsilon}_i\dot{\epsilon}_j + 2\sum_{\substack{i,j=n+1\\ i<j}}^{N} d_{ij}(\boldsymbol{\epsilon})\dot{\epsilon}_i\dot{\epsilon}_j\right. \\
&\qquad \left. + 2\sum_{i=1}^{n-1}\sum_{j=n+1}^{N} e_{ij}(\boldsymbol{\epsilon})\dot{\epsilon}_i\dot{\epsilon}_j\right\}
\end{aligned}$$

where

$$a_j^{(n)}(\boldsymbol{\epsilon}) := \begin{cases} 3(L+v_j)^2 & \text{if} j \le n-1 \\ L^2 + 3Lv_n + 3v_n^2 & \text{if} j = n \\ 3v_j^2 & \text{if} j \ge n+1 \end{cases}, \qquad b_j^{(n)}(\boldsymbol{\epsilon}) := \begin{cases} 3(L+v_j)(\frac{L}{2}+v_n) & \text{if} j \le n-1 \\ 3v_j(\frac{L}{2}+v_n) & \text{if} j \ge n+1 \end{cases},$$

$$c_{ij}(\boldsymbol{\epsilon}) := 3(L+v_i)(L+v_j)\,, \quad d_{ij}(\boldsymbol{\epsilon}) := 3L + v_iv_j\,, \quad \text{and} \quad e_{ij}(\boldsymbol{\epsilon}) := 3(L+v_i)v_j\,.$$

Then

$$D_n = \frac{L}{3}\left(1+\epsilon_n\right)^{1-p}\left[\dot{\boldsymbol{\epsilon}}\cdot\mathbb{D}_n(\boldsymbol{\epsilon})\dot{\boldsymbol{\epsilon}}\right]$$

where, for $n = 1, \dots, N$,

$$\mathbb{D}_n(\boldsymbol{\epsilon}) := \begin{bmatrix} a_1^{(n)} & c_{12} & \cdots & c_{1,n-1} & b_1^{(n)} & e_{1,n+1} & \cdots & \cdots & e_{1,N} \\ c_{12} & \ddots & \ddots & \vdots & \vdots & \vdots & & & \vdots \\ \vdots & \ddots & \ddots & c_{n-2,n-1} & \vdots & \vdots & & & \vdots \\ c_{1,n-1} & \cdots & c_{n-2,n-1} & a_{n-1}^{(n)} & b_{n-1}^{(n)} & e_{n-1,n+1} & \cdots & \cdots & e_{n-1,N} \\ b_1^{(n)} & \cdots & \cdots & b_{n-1}^{(n)} & a_n^{(n)} & b_{n+1}^{(n)} & \cdots & \cdots & b_N^{(n)} \\ e_{1,n+1} & \cdots & \cdots & e_{n-1,n+1} & b_{n+1}^{(n)} & a_{n+1}^{(n)} & d_{n+1,n+2} & \cdots & d_{n+1,N} \\ \vdots & & & \vdots & \vdots & d_{n+1,n+2} & \ddots & \ddots & \vdots \\ \vdots & & & \vdots & \vdots & \vdots & \ddots & \ddots & d_{N-1,N} \\ e_{1,N} & \cdots & \cdots & e_{n-1,N} & b_N^{(n)} & d_{n+1,N} & \cdots & d_{N-1,N} & a_N^{(n)} \end{bmatrix} .$$

Thus

$$d_1(t, \boldsymbol{\epsilon}, \dot{\boldsymbol{\epsilon}}) = \sum_{n=1}^{N} D_n = \sum_{n=1}^{N} \frac{L}{3} (1 + \epsilon_n)^{1-p} [\dot{\boldsymbol{\epsilon}} \cdot \mathbb{D}_n(\boldsymbol{\epsilon})\dot{\boldsymbol{\epsilon}}] = \dot{\boldsymbol{\epsilon}} \cdot \mathbb{D}(\boldsymbol{\epsilon})\dot{\boldsymbol{\epsilon}}$$

where

$$\mathbb{D}(\boldsymbol{\epsilon}) := \frac{L}{3} \sum_{n=1}^{N} (1 + \epsilon_n)^{1-p} \mathbb{D}_n(\boldsymbol{\epsilon}) . \tag{12}$$

C2. Operator $\mathbb{G}$

We recall that, by definition,

$$\mathbb{G} := \mathbb{D}(\mathbf{0}) + w\mathbb{I}_N = \frac{L}{3} \sum_{n=1}^{N} \mathbb{D}_n(\mathbf{0}) + w\mathbb{I}_N .$$

From (1)

$$v_n(\mathbf{0}) = -\frac{L}{2} \frac{2(N-n)+1}{N} \quad \text{for } n = 1, \dots, N ,$$

and hence

$$a_j^{(n)}(\mathbf{0}) = \begin{cases} \frac{3L^2}{4N^2}(2j-1)^2 & \text{if } j < n \\ \frac{3L^2}{4N^2}\left[\frac{4}{3}N^2 + 2N(1-2n) + 4n(n-1) + 1\right] & \text{if } j = n \\ \frac{3L^2}{4N^2}\left[2(N-j)+1\right]^2 & \text{if } j > n \end{cases} ,$$

$$b_j^{(n)}(\mathbf{0}) = \begin{cases} \frac{3L^2}{4N^2}(2j-1)(2n-N-1) & \text{if } j < n \\ \frac{3L^2}{4N^2}(2N-2j+1)(N-2n+1) & \text{if } j > n \end{cases} ,$$

$$c_{ij}(\mathbf{0}) = \frac{3L^2}{4N^2}(2j-1)(2i-1) ,$$

$$d_{ij}(\mathbf{0}) = \frac{3L^2}{4N^2}\left[2(N-i)+1\right]\left[2(N-j)+1\right] ,$$

$$e_{ij}(\mathbf{0}) = \frac{3L^2}{4N^2}(2i-1)\left[2(j-N)-1\right] .$$

Since

$$\mathbb{D}(\mathbf{0}) := \frac{L}{3}\sum_{n=1}^{N}\mathbb{D}_n(\mathbf{0}) = \frac{L^3}{4N^2}\sum_{n=1}^{N}\tfrac{4N^2}{3L^2}\mathbb{D}_n(\mathbf{0})$$

we get

- for $i<j$,

$$\begin{aligned}\{\mathbb{D}(\mathbf{0})\}_{ij} &= \frac{L^3}{4N^2}\Bigg[\sum_{n=1}^{i-1}(2N-2i+1)\,(2N-2j+1)+(2j-2N-1)\,(2i-N-1)\\ &\quad+\sum_{n=i+1}^{j-1}(2i-1)\,(2j-2N-1)+(2i-1)(2j-N-1)+\sum_{n=j+1}^{N}(2i-1)\,(2j-1)\Bigg]\\ &=\frac{L^3}{4N}(2i-1)\,[2(N-j)+1]\,,\end{aligned}$$

- for $i=j$,

$$\begin{aligned}\{\mathbb{D}(\mathbf{0})\}_{ii} &= \frac{L^3}{4N^2}\left[\sum_{n=1}^{i-1}(2N-2i+1)^2+\left(\frac{4}{3}N^2+2N(1-2i)+4i(i-1)+1\right)+\sum_{n=i+1}^{N}(2i-1)^2\right]\\ &=\frac{L^3}{12N}\left[4N(3i-2)-3(2i-1)^2\right],\end{aligned}$$

- for $i>j$, by symmetry,

$$\{\mathbb{D}(\mathbf{0})\}_{ij} = \{\mathbb{D}(\mathbf{0})\}_{ji} = \frac{L^3}{4N}(2j-1)\,[2(N-i)+1]\,.$$

Therefore,

$$\{\mathbb{G}\}_{ij} = \begin{cases}\frac{L^3}{4N}(2i-1)\,(2(N-j)+1) & \text{if } i<j\\ \frac{L^3}{12N}\left[4N(3i-2)-3\,(2i-1)^2\right]+w & \text{if } i=j\\ \frac{L^3}{4N}(2j-1)\,(2(N-i)+1) & \text{if } i>j\end{cases}. \tag{13}$$

Notice that $\mathbb{G}$ is symmetric both about the main diagonal (by construction) and about the secondary diagonal indeed

- for $i<j$,

$$\begin{aligned}\{\mathbb{G}\}_{ij} &= \frac{L^3}{4N}(2i-1)\,(2(N-j)+1) = \frac{L^3}{4N}(2(N+1-j)-1)\,(2(N-(N+1-i))+1)\\ &= \{\mathbb{G}\}_{(N+1-j)(N+1-i)}\end{aligned}$$

- for $i = j$,

$$\begin{aligned}\{\mathbb{G}\}_{ii} &= \frac{L^3}{12N}\left[4N(3i-2) - 3\,(2i-1)^2\right] + w \\ &= \frac{L^3}{12N}\left[4N(3(N+1-i)-2) - 3\,(2(N+1-i)-1)^2\right] + w \\ &= \{\mathbb{G}\}_{(N+1-i)(N+1-i)}\end{aligned}$$

- for $i > j$, by symmetry,

$$\{\mathbb{G}\}_{ij} = \{\mathbb{G}\}_{ji} = \{\mathbb{G}\}_{(N+1-i)(N+1-j)} = \{\mathbb{G}\}_{(N+1-j)(N+1-i)}\ .$$

Such a property is usually referred to as “bisymmetry” and it implies “centrosymmetry”, i.e.,, symmetry about the center or, in other terms,

$$\{\mathbb{G}\}_{ij} = \{\mathbb{G}\}_{(N+1-i)(N+1-j)} \qquad \forall i,j = 1,\ldots,N\ .$$

C3. Optimal control problem for the periodic version

Consider the optimal control problem

$$\begin{aligned}&\max_{\boldsymbol{\epsilon}\in\mathcal{S}_u^\star} \quad V[\mathbf{u},\dot{\mathbf{u}}] := \int_0^T \dot{\mathbf{u}}\cdot\mathbb{V}_u^\star\mathbf{u}\,dt \\ &\mathcal{S}_u^\star := \left\{\mathbf{u}\in C^3\left(\mathbb{R},\mathbb{R}^N\right)\ \middle|\ \mathbf{u}(0)=\mathbf{u}(T) \quad \wedge \quad E\left[\mathbf{u},\dot{\mathbf{u}}\right] := \int_0^T \dot{\mathbf{u}}\cdot\mathbb{G}_u^\star\dot{\mathbf{u}}\,dt = c\right\}\end{aligned} \tag{14}$$

where

$$\mathbb{V}_u^\star := J_{per}^T\mathbb{V}J_{per}\ , \quad \mathbb{G}_u^\star := J_{per}^T\mathbb{G}J_{per}\ , \quad \boldsymbol{\epsilon} = \mathbb{J}_{per}\boldsymbol{u}$$

and

$$\mathbb{J}_{per} := \frac{1}{L}\begin{bmatrix} 1 & & & -1 \\ -1 & 1 & & \\ & \ddots & \ddots & \\ & & -1 & 1 \end{bmatrix}$$

In view of (2) and (13), some calculations lead to

$$\{\mathbb{V}_u^\star\}_{ij} = \begin{cases} \frac{p-1}{2NL} & \text{if } i=j-1 \text{ or } (i,j)=(N,1) \\ -\frac{p-1}{2NL} & \text{if } i=j+1 \text{ or } (i,j)=(1,N) \\ 0 & \text{else} \end{cases} \tag{15}$$

and

$$\{\mathbb{G}_u^\star\}_{ij} = \begin{cases} \frac{2N-3}{3N}L + \frac{2w}{L^2} & \text{if } i=j-1 \text{ or } (i,j)=(N,1) \\ \frac{N-6}{6N}L - \frac{w}{L^2} & \text{if } i=j+1 \text{ or } (i,j)=(1,N) \\ -\frac{L}{N} & \text{else} \end{cases}\ . \tag{16}$$

Hence, Euler-Lagrange equations associated with (14), are given by

$$\mathbb{V}_u^\star \dot{\boldsymbol{u}} = \lambda \, \mathbb{G}_u^\star \ddot{\boldsymbol{u}} \tag{17}$$

where $\mathbb{V}_u^\star$ and $\mathbb{G}_u^\star$ are circulant and, for this reason, diagonalizable on a common orthonormal basis, which is called *Fourier basis.* Indeed,

$$\mathbb{V}_u^\star = \{\mathbb{V}_u^\star\}_{1,1} \, \mathbb{I}_N + \{\mathbb{V}_u^\star\}_{1,2} \, \mathbb{E} + \cdots + \{\mathbb{V}_u^\star\}_{1,N} \, \mathbb{E}^{N-1}$$

$$\mathbb{G}_u^\star = \{\mathbb{G}_u^\star\}_{1,1} \, \mathbb{I}_N + \{\mathbb{G}_u^\star\}_{1,2} \, \mathbb{E} + \cdots + \{\mathbb{G}_u^\star\}_{1,N} \, \mathbb{E}^{N-1}$$

where

$$\mathbb{E} := \begin{bmatrix} & 1 & & & \\ & & 1 & & \\ & & & \ddots & \\ & & & & 1 \\ 1 & & & & \end{bmatrix} \qquad \left(\mathbb{E}^N = \mathbb{I}_N\right),$$

whose eigenvectors are

$$\mathbf{e}_j = \frac{1}{N} \begin{bmatrix} 1 \\ e^{i\frac{2\pi}{N}(j-1)} \\ \vdots \\ e^{i\frac{2\pi}{N}(j-1)(N-1)} \end{bmatrix} \quad \left(\text{corresponding to the eigenvalue } \mu_j = e^{i\frac{2\pi}{N}(j-1)}\right) \text{ for } j = 1, \ldots, N .$$

Therefore

$$\mathbb{G}_u^\star \mathbf{e}_j = g_j \mathbf{e}_j \quad \text{where} \quad g_j := \left(\sum_{k=1}^{N} \{\mathbb{G}_u^\star\}_{1,k} \, \mu_j^{k-1} \right) \tag{18}$$

and

$$\mathbb{V}_u^\star \mathbf{e}_j = v_j \mathbf{e}_j \quad \text{where} \quad v_j := \left(\sum_{k=1}^{N} \{\mathbb{V}_u^\star\}_{1,k} \, \mu_j^{k-1} \right) . \tag{19}$$

Writing

$$\mathbf{u}(t) = \sum_{j=1}^{N} u_j(t) \mathbf{e}_j$$

we can project equation (17) along the eigenvectors, i.e.,

$$\lambda g_j \ddot{u}_j(t) = v_j \dot{u}_j(t) \qquad \forall j .$$

Thus, up to a constant,

$$u_j(t) = \begin{cases} \frac{\alpha_j \lambda \sqrt{g_j}}{v_j} e^{\frac{v_j}{\lambda g_j} t} & \text{for } j = 1, \ldots, N \text{ s.t. } g_j, v_j \neq 0 \\ \alpha_j t & \text{else} \end{cases}$$

where α_j are complex constants; furthermore, periodicity yields

$$\begin{cases} \lambda = \frac{T}{2\pi k_j}\frac{v_j}{ig_j} & \text{for } j = 1,\dots,N \text{ s.t. } g_j, v_j \neq 0 \\ \alpha_j = 0 & \text{else} \end{cases}$$

where $k_j \in \mathbb{N}\ \forall j$.

On the other hand,

$$V[\boldsymbol{u},\dot{\boldsymbol{u}}] = -\int_0^T \mathbb{V}_u^\star \dot{\boldsymbol{u}} \cdot \boldsymbol{u}\, dt = -\int_0^T \lambda \mathbb{G}_u^\star \ddot{\boldsymbol{u}} \cdot \boldsymbol{u}\, dt = \lambda \int_0^T \mathbb{G}_u^\star \dot{\boldsymbol{u}} \cdot \dot{\boldsymbol{u}}\, dt = \lambda E\left[\boldsymbol{u},\dot{\boldsymbol{u}}\right]$$

where $E\left[\boldsymbol{u},\dot{\boldsymbol{u}}\right] = c$ is constrained by the optimization problem. Then maximizing the approximated displacement leads to take λ as big as possible i.e.,

$$\lambda = \frac{T}{2\pi}\frac{v_M}{ig_M} \quad \text{where } \frac{v_M}{ig_M} = \max_{\substack{j=1,\dots,N \\ g_j,\, v_j \neq 0}} \frac{v_j}{ig_j}$$

and, in order to preserve the periodicity,

$$\alpha_j = 0 \qquad \text{for } j \notin \left\{ k \in \{1,\dots,N\} : \frac{v_k}{ig_k} = \max_{\substack{j=1,\dots,N \\ g_j,\, v_j \neq 0}} \left| \frac{v_j}{ig_j} \right| \right\}.$$

In particular, in view of (18) and (19), we have

$$v_j = \sum_{k=1}^{N} \{\mathbb{V}_u^\star\}_{1,k}\, e^{i\frac{2\pi}{N}(k-1)(j-1)} = \frac{(p-1)}{2NL}\left[e^{i\frac{2\pi}{N}(j-1)} - e^{-i\frac{2\pi}{N}(j-1)}\right] = i\,\frac{(p-1)}{NL}\sin\left(\frac{2\pi(j-1)}{N}\right)$$

and

$$\begin{aligned} g_j &= \sum_{k=1}^{N} \{\mathbb{G}_u^\star\}_{1,k}\, e^{i\frac{2\pi}{N}(k-1)(j-1)} \\ &= \{\mathbb{G}_u^\star\}_{1,1} + \{\mathbb{G}_u^\star\}_{1,2}\left[e^{i\frac{2\pi}{N}(j-1)} - e^{i\frac{2\pi}{N}(j-1)(N-1)}\right] + \{\mathbb{G}_u^\star\}_{1,3}\sum_{k=3}^{N-1} e^{i\frac{2\pi}{N}(j-1)(k-1)} \\ &= \begin{cases} 2\left[\frac{L}{3} + \frac{w}{L^2} + \left(\frac{L}{6} - \frac{w}{L^2}\right)\cos\left(\frac{2\pi(j-1)}{N}\right)\right] & \text{for } j \neq 1 \\ 0 & \text{for } j = 1 \end{cases}. \end{aligned}$$

Notice that

$$\frac{v_j}{g_j} = -\frac{v_{N-j+2}}{g_{N-j+2}} \quad \text{for } j = 2,\dots,N$$

and hence, a (real) solution has the form (up to a constant)

$$\mathbf{u}(t) = \frac{\alpha T}{2\pi i\sqrt{g_M}} e^{\frac{2\pi i}{T}t}\mathbf{e}_M - \frac{\bar{\alpha} T}{2\pi i\sqrt{g_M}} e^{-\frac{2\pi i}{T}t}\bar{\mathbf{e}}_M = -\frac{T}{\pi\sqrt{g_M}}\Re\left(\alpha i e^{\frac{2\pi i}{T}t}\mathbf{e}_M\right)$$

where $\alpha \in \mathbb{C} \setminus \{0\}$ fulfills the constraint

$$||\alpha|| = \sqrt{\frac{c}{2T}} \qquad \left(\text{since } \int_0^T \mathbb{G}_u^\star \dot{\boldsymbol{u}} \cdot \dot{\boldsymbol{u}}\, dt = 2T||\alpha||^2\right)$$

and

$$\mathbf{e}_M := \frac{1}{N} \begin{bmatrix} 1 \\ e^{i\frac{2\pi}{N}(M-1)} \\ \vdots \\ e^{i\frac{2\pi}{N}(M-1)(N-1)} \end{bmatrix}.$$

In terms of strains,

$$\begin{cases} \epsilon_1 = \dfrac{u_1 - u_N}{L} = -\dfrac{T}{\pi N L \sqrt{g_M}} \Re\left(\alpha i e^{\frac{2\pi i}{T}t}\left[1 - e^{-i\frac{2\pi}{N}(M-1)}\right]\right) \\ \epsilon_j = \dfrac{u_j - u_{j-1}}{L} = -\dfrac{T}{\pi N L \sqrt{g_M}} \Re\left(\alpha i e^{\frac{2\pi i}{T}t}\left[1 - e^{-i\frac{2\pi}{N}(M-1)}\right] e^{i\frac{2\pi}{N}(M-1)(j-1)}\right) \quad \text{for } j = 2, \ldots, N \end{cases}$$

whence the exact peristalsis

$$\boldsymbol{\epsilon}(t) = -\frac{T}{\pi\sqrt{g_M}} \Re\left(\alpha i e^{\frac{2\pi i}{T}t}\mathbf{e}\right) \quad \text{where} \quad \mathbf{e} := \begin{bmatrix} e_1 \\ e^{i\frac{2\pi(M-1)}{N}} e_1 \\ \vdots \\ e^{i\frac{2\pi(M-1)}{N}(n-1)} e_1 \\ \vdots \\ e^{i\frac{2\pi(M-1)}{N}(N-1)} e_1 \end{bmatrix}, \; e_1 := \frac{1}{NL}\left[1 - e^{-i\frac{2\pi}{N}(M-1)}\right].$$

Finally, observe that the wavenumber (i.e., the frequency in space) of the peristalsis is $k = M - 1$ and it is the result of

$$\max_{k=1,\ldots N-1} \frac{v_{k+1}}{i g_{k+1}} = \max_{k=1,\ldots N-1} \frac{\frac{(p-1)}{NL}\sin\left(\frac{2\pi k}{N}\right)}{\left[\frac{L}{3} + \frac{w}{L^2} + \left(\frac{L}{6} - \frac{w}{L^2}\right)\cos\left(\frac{2\pi k}{N}\right)\right]}$$

i.e.,

$$k \sim \frac{N}{2\pi} \arccos\left(\frac{1}{2}\frac{6w - L^3}{3w + L^3}\right).$$

Notice that

- for $w \to +\infty$, the wavenumber k tends to 1;
- for $w = 0$, the wavenumber k gets close to $\frac{N}{3}$.

APPENDIX D
PROOF OF REFLECTIONAL SYMMETRY

Consider the optimization problem

$$\max_{\boldsymbol{\eta}\in[0,2\pi)^N} \quad u_s(\boldsymbol{\eta}) := \frac{1}{T}\int_0^T \mathbf{v}(\boldsymbol{\epsilon}) \cdot \dot{\boldsymbol{\epsilon}}\, dt \tag{20}$$

where, for $n = 1, \ldots, N$,

$$\epsilon_n(t) := a\sin\left(\tfrac{2\pi}{T}t + \eta_n\right) \qquad \text{and} \qquad v_n(t) := -\frac{L}{2}\frac{(1+\epsilon_n)^{1-p} + 2\sum\limits_{i=n+1}^{N}(1+\epsilon_i)^{1-p}}{\sum\limits_{j=1}^{N}(1+\epsilon_j)^{1-p}}\,.$$

Assume that (20) has a unique solution and denote it by

$$\boldsymbol{\epsilon}(\boldsymbol{\eta})(t) = \left\{\epsilon_n(t) = a\sin\left(\tfrac{2\pi}{T}t + \eta_n\right)\right\}_{n=1,\ldots,N}\,.$$

Consider

$$\tilde{\boldsymbol{\epsilon}}(t) := \tilde{\boldsymbol{\epsilon}}(\tilde{\boldsymbol{\eta}})(t) \qquad \text{where} \quad \tilde{\boldsymbol{\eta}} = -\mathbb{K}\boldsymbol{\eta} + 2\pi\,, \quad \mathbb{K} := \begin{bmatrix} 0 & 0 & \cdots & 0 & 1 \\ 0 & 0 & \cdots & 1 & 0 \\ \vdots & \vdots & \iddots & \vdots & \vdots \\ 0 & 1 & \cdots & 0 & 0 \\ 1 & 0 & \cdots & 0 & 0 \end{bmatrix} \in \mathbb{R}^{N\times N}\,.$$

Notice that for $n = 1, \ldots, N$,

$$\tilde{\epsilon}_n(t) = a\sin\left(\tfrac{2\pi}{T}t - (\mathbb{K}\eta)_n\right) = \{-\mathbb{K}\epsilon(-t)\}_n = -\epsilon_{N+1-n}(-t)$$

and, consequently,

$$\dot{\tilde{\epsilon}}_n(t) = \dot{\epsilon}_{N+1-n}(-t)\,.$$

Since both $\boldsymbol{\epsilon}(t)$ and $\dot{\boldsymbol{\epsilon}}(t)$ are periodic functions of period T, we have

$$\begin{aligned} -\frac{2}{L}\int_0^T \mathbf{v}(\tilde{\boldsymbol{\epsilon}}) \cdot \dot{\tilde{\boldsymbol{\epsilon}}}\, dt &= \int_0^T \sum_{n=1}^{N} \dot{\tilde{\epsilon}}_n \left[\left(\sum_{j=1}^{N}(1+\tilde{\epsilon}_j)^{1-p}\right)^{-1}\left((1+\tilde{\epsilon}_n)^{1-p} + 2\sum_{i=n+1}^{N}(1+\tilde{\epsilon}_i)^{1-p}\right)\right] dt \\ &= \int_0^T \sum_{n=1}^{N} \dot{\epsilon}_{N+1-n}(-t) \left[\left(\sum_{j=1}^{N}(1-\epsilon_{N+1-j}(-t))^{1-p}\right)^{-1}\Bigg((1-\epsilon_{N+1-n}(-t))^{1-p}\right. \\ &\quad \left. + 2\sum_{i=n+1}^{N}(1-\epsilon_{N+1-i}(-t))^{1-p}\Bigg)\right] dt \end{aligned}$$

$$
\begin{aligned}
&= \int_0^T \sum_{n=1}^N \dot{\epsilon}_{N+1-n}(t) \left[\left(\sum_{j=1}^N (1-\epsilon_{N+1-j}(t))^{1-p} \right)^{-1} \left((1-\epsilon_{N+1-n}(t))^{1-p} \right.\right. \\
&\quad \left.\left. +2 \sum_{i=n+1}^N (1-\epsilon_{N+1-i}(t))^{1-p} \right) \right] dt \\
&= \int_0^T \sum_{n=1}^N \dot{\epsilon}_n \left[\left(\sum_{j=1}^N (1-\epsilon_j)^{1-p} \right)^{-1} \left((1-\epsilon_n)^{1-p} + 2 \sum_{i=1}^{n-1} (1-\epsilon_i)^{1-p} \right) \right] dt \\
&= -\frac{2}{L} \int_0^T \mathbf{v}^\star(\boldsymbol{\epsilon}) \cdot \dot{\boldsymbol{\epsilon}}\, dt
\end{aligned}
$$

where

$$
v_n^\star := -\frac{L}{2} \frac{(1-\epsilon_n)^{1-p} + 2 \sum_{i=1}^{n-1} (1-\epsilon_i)^{1-p}}{\sum_{j=1}^N (1-\epsilon_j)^{1-p}} .
$$

Observe that the last integral can be rewritten as

$$
\int_0^T \mathbf{v}^\star(\boldsymbol{\epsilon}) \cdot \dot{\boldsymbol{\epsilon}}\, dt = \oint_{\partial\Omega} \omega^\star
$$

where $\partial\Omega$ is the closed curve described by $\boldsymbol{\epsilon}(t)$ and $\omega^\star$ is the 1-form given by

$$
\omega^\star := \sum_{n=1}^N v_n^\star \, \mathrm{d}\epsilon_n . \tag{21}
$$

The exterior derivative of (21) is the following 2-form

$$
\mathrm{d}\omega^\star = \sum_{\substack{i,j=1 \\ i \neq j}}^N \frac{\partial v_i^\star}{\partial \epsilon_j} \mathrm{d}\epsilon_j \wedge \mathrm{d}\epsilon_i = \sum_{\substack{i,j=1 \\ i < j}}^N A_{ij}^\star(\boldsymbol{\epsilon}) \mathrm{d}\epsilon_i \wedge \mathrm{d}\epsilon_j \qquad \text{where} \qquad A_{ij}^\star(\boldsymbol{\epsilon}) := \left(\frac{\partial v_j^\star}{\partial \epsilon_i} - \frac{\partial v_i^\star}{\partial \epsilon_j} \right) .
$$

In particular, since

$$
\frac{\partial v_i^\star}{\partial \epsilon_j} = \begin{cases} -\left[\sum_{n=1}^N (1-\epsilon_n)^{1-p} \right]^{-2} (1-p)(1-\epsilon_j)^{-p} \left[(1-\epsilon_i)^{1-p} + 2 \sum_{n=i+1}^N (1-\epsilon_n)^{1-p} \right] & j < i \\ \left[\sum_{n=1}^N (1-\epsilon_n)^{1-p} \right]^{-2} (1-p)(1-\epsilon_j)^{-p} \left[(1-\epsilon_i)^{1-p} + 2 \sum_{n=1}^{i-1} (1-\epsilon_n)^{1-p} \right] & j > i \end{cases}
$$

we get

$$
\begin{aligned}
A^{\star}_{ij}(\boldsymbol{\epsilon}) = &-\left[\sum_{n=1}^{N}(1-\epsilon_n)^{1-p}\right]^{-2}(1-p)\left[(1-\epsilon_i)^{-p}\left((1-\epsilon_j)^{1-p}+2\sum_{n=j+1}^{N}(1-\epsilon_n)^{1-p}\right)\right.\\
&\left.+(1-\epsilon_j)^{-p}\left((1-\epsilon_i)^{1-p}+2\sum_{n=1}^{i-1}(1-\epsilon_n)^{1-p}\right)\right].
\end{aligned}
$$

Therefore, by Stokes' theorem (see any differential geometry textbook, e.g., McInerney (2013)),

$$
\int_0^T \mathbf{v}^{\star}(\boldsymbol{\epsilon})\cdot\dot{\boldsymbol{\epsilon}}\,dt = \oint_{\partial\Omega}\omega^{\star} = \int_{\Omega}\mathrm{d}\omega^{\star} = \int_{\Omega}\sum_{i<j}A^{\star}_{ij}\,\mathrm{d}\epsilon_i\wedge\mathrm{d}\epsilon_j
$$

and, since the domain Ω is invariant with respect to the reflection about the origin,

$$
\int_{\Omega}\sum_{i<j}A^{\star}_{ij}\,\mathrm{d}\epsilon_i\wedge\mathrm{d}\epsilon_j = \int_{\Omega}\sum_{i<j}A^{\star}_{ij}(-\boldsymbol{\epsilon})\,\mathrm{d}\epsilon_i\wedge\mathrm{d}\epsilon_j\,.
$$

Similarly, the exterior derivative of

$$
\omega := \sum_{n=1}^{N} v_n\,\mathrm{d}\epsilon_n\,,
$$

is given by

$$
\mathrm{d}\omega = \sum_{\substack{i,j=1\\ i<j}}^{N} A_{ij}(\boldsymbol{\epsilon})\mathrm{d}\epsilon_i\wedge\mathrm{d}\epsilon_j \qquad \text{where} \qquad A_{ij}(\boldsymbol{\epsilon}) := \left(\frac{\partial v_j}{\partial\epsilon_i}-\frac{\partial v_i}{\partial\epsilon_j}\right).
$$

Since

$$
\frac{\partial v_i}{\partial\epsilon_j} = \begin{cases}
-\left[\sum\limits_{n=1}^{N}(1+\epsilon_n)^{1-p}\right]^{-2}(1-p)(1+\epsilon_j)^{-p}\left[(1+\epsilon_i)^{1-p}+2\sum\limits_{n=i+1}^{N}(1+\epsilon_n)^{1-p}\right] & j<i\\
\left[\sum\limits_{n=1}^{N}(1+\epsilon_n)^{1-p}\right]^{-2}(1-p)(1+\epsilon_j)^{-p}\left[(1+\epsilon_i)^{1-p}+2\sum\limits_{n=1}^{i-1}(1+\epsilon_n)^{1-p}\right] & j>i
\end{cases},
$$

we notice that

$$
\begin{aligned}
A_{ij}(\boldsymbol{\epsilon}) = &-\left[\sum_{n=1}^{N}(1-\epsilon_n)^{1-p}\right]^{-2}(1-p)\left[(1-\epsilon_i)^{-p}\left((1-\epsilon_j)^{1-p}+2\sum_{n=j+1}^{N}(1-\epsilon_n)^{1-p}\right)\right.\\
&\left.+(1-\epsilon_j)^{-p}\left((1-\epsilon_i)^{1-p}+2\sum_{n=1}^{i-1}(1-\epsilon_n)^{1-p}\right)\right]\\
= &\,A^{\star}_{ij}(-\boldsymbol{\epsilon})\,.
\end{aligned}
$$

Therefore,

$$
\int_0^T \mathbf{v}^{\star}(\boldsymbol{\epsilon})\cdot\dot{\boldsymbol{\epsilon}}\,dt = \oint_{\partial\Omega}\omega^{\star} = \int_{\Omega}\mathrm{d}\omega^{\star} = \int_{\Omega}\mathrm{d}\omega = \oint_{\partial\Omega}\omega = \int_0^T \mathbf{v}(\boldsymbol{\epsilon})\cdot\dot{\boldsymbol{\epsilon}}\,dt
$$

whence we conclude

$$\frac{1}{T}\int_0^T \mathbf{v}(\tilde{\boldsymbol{\epsilon}}) \cdot \dot{\tilde{\boldsymbol{\epsilon}}}\, dt = \frac{1}{T}\int_0^T \mathbf{v}(\boldsymbol{\epsilon}) \cdot \dot{\boldsymbol{\epsilon}}\, dt\,.$$

In other terms, $\tilde{\boldsymbol{\epsilon}}$ is a solution to (20) and, by uniqueness of the solution,

$$\epsilon_n(t) = -\epsilon_{N+1-n}(-t)\,.$$

Then

$$\boldsymbol{\eta} = -\mathbb{K}\boldsymbol{\eta} + 2\pi$$

which leads to the "reflectional symmetry about the center", namely,

$$\eta_{n+1} - \eta_n = \eta_{N+1-n} - \eta_{N-n} \qquad \forall\, n\,.$$
